# Integrated Functional and scRNA-Seq Analyses Reveal Convergence of M-CSF– and GM-CSF–Derived Macrophages Following IL-27 Polarization

**DOI:** 10.3390/cells15060528

**Published:** 2026-03-16

**Authors:** Tomozumi Imamichi, Jun Yang, Qian Chen, Udeshika Kariyawasam, Mayra Marquez, Jeanette Higgins, Jordan Metz, Homa Nath Sharma, Michael W. Baseler, Hongyan Sui

**Affiliations:** 1 Laboratory of Human Retrovirology and Immunoinformatics, Frederick National Laboratory for Cancer Research, Fredrick, MD 21702, USA; jyang@nih.gov (J.Y.); chenq3@nih.gov (Q.C.); kariyawasamul@nih.gov (U.K.); marquezm3@nih.gov (M.M.); hongyan.sui@nih.gov (H.S.); 2 AIDS Monitoring Laboratory, Frederick National Laboratory for Cancer Research, Fredrick, MD 21702, USAmetzmj@nih.gov (J.M.);

**Keywords:** IL-27, convergence, polarization, scRNA Seq, M-CSF-differentiated MDMs, GM-CSF-differentiated MDMs

## Abstract

**Highlights:**

**What are the main findings?**

- Two distinct macrophage subsets—M-CSF– and GM-CSF–differentiated monocyte-derived macrophages (MDMs)—are reprogrammed by IL-27 into a convergent phenotype

**What are the implications of the main findings?**

- IL-27 functions as a convergent polarization cytokine.
- IL-27 may play a central role in regulating macrophage plasticity.

**Abstract:**

Macrophages differentiated with macrophage colony-stimulating factor (M-CSF) (M-Mac) are widely used as an experimental model. Interleukin 27 (IL-27)-polarized M-Mac (27M-Mac) suppresses HIV replication; however, the effects of IL-27 polarization on granulocyte-macrophage colony-stimulating factor (GM-CSF)-induced macrophages (GM-Mac) remain less investigation. Here, we compare multiple functional properties and gene expression profiles of 27M-Mac and IL-27-polarized GM-Mac (27GM-Mac). M-Mac and GM-Mac were generated from monocytes of healthy donors and subsequently treated with IL-27 for three days. HIV replication in 27M-Mac, GM-Mac, and 27GM-Mac was suppressed to nearly 10% of that in M-Mac; however, single-cell RNA sequencing showed that M-Mac clustered with GM-Mac, and 27M-Mac clustered with 27GM-Mac. Expression of CD38 and secretion of CXCL9 and C1q were significantly increased in 27M-Mac and 27GM-Mac compared with M-Mac and GM-Mac. Although CD16 and CD64 expression increased in 27M-Mac and 27GM-Mac relative to their respective controls, phagocytic activity in 27M-Mac and 27GM-Mac was 30% of that in M-Mac. Autophagy was promoted 3.7-fold more strongly in 27M-Mac than in M-Mac, reaching levels comparable to those in GM-Mac and 27GM-Mac. Collectively, these findings indicate that IL-27 polarizes M-Mac and GM-Mac toward transcriptionally and functionally similar subtypes, providing insight into the role of IL-27 in macrophage polarization and plasticity.

## 1. Introduction

Interleukin (IL)-27 is a heterodimeric cytokine that belongs to the IL-12 family based on structural similarity and to the IL-6/gp130 family based on receptor usage [1,2]. IL-27 was initially identified as a modifier of T-cell function, but subsequent studies have demonstrated its multifaceted role in numerous cell types, including neutrophils, macrophages, dendritic cells, hepatocytes, and keratinocytes [3,4,5,6,7,8]. IL-27 signals through the IL-27 receptor (IL-27R), which comprises gp130 and WSX-1 [2,9]. IL-27 was identified as an anti-HIV factor in the culture supernatants from peripheral blood mononuclear cells (PBMCs) treated with a cervical cancer vaccine [4], and it was subsequently shown to suppress HIV replication in T cells, macrophages, and dendritic cells [10,11]. IL-27 also inhibits infection by multiple other viruses, including the influenza virus [12], hepatitis C virus [7], hepatitis B virus [13,14], cytomegalovirus [15], coxsackievirus B3, respiratory syncytial virus [16], dengue virus [17], chikungunya virus [18], Zika virus [19], and the Mayaro virus [20]. Recent studies suggest that IL-27 may contribute to the suppression of SARS-CoV-2 infection [21,22].

The antiviral activity of IL-27 is mediated by the induction of multiple interferon-stimulated genes (ISGs) [10,18,20,23] via activation of STAT1 and STAT3 [10,11]. ISG induction by IL-27 is cell type dependent: in some contexts, it requires type I interferon (IFN) (IFN-α or IFN-β) [23], whereas in others it is IFN independent [10,24]. Accordingly, IL-27 has been proposed as an immunotherapeutic agent for viral infections [25] and other diseases [26]. However, in vitro studies of IL-27 antiviral activity in human primary macrophages typically do not account for macrophage subset heterogeneity. Macrophages are commonly differentiated from peripheral blood monocytes using macrophage colony-stimulating factor (M-CSF) [4,12,17,27], human serum [4,28,29], or fetal bovine serum (FBS) [23], and the resulting monocyte-derived macrophages (MDMs) are used as virus-permissive host cells. Given macrophage diversity, distinct subsets can differ substantially in function and activity.

Macrophages act as phagocytes, generate reactive oxygen species (ROS), exhibit chemotaxis, secrete cytokines and chemokines, present antigens to support adaptive immune response, and secrete type-I and type-III IFNs in response to various pathogens in the innate immunity [30,31,32,33,34,35,36,37]. Macrophages are often categorized as M1 (classically activated, pro-inflammatory) or M2 (alternatively activated, anti-inflammatory) [38,39,40]. In addition, several other macrophage subsets have been described, including regulatory macrophages (Mreg), heme-related macrophages (Mhem), oxidized macrophages (Mox), M3 macrophages, and CD169^+^/TCR^+^ macrophages [41,42,43,44,45,46,47,48,49,50,51,52]. Monocyte-derived macrophages (MDMs) induced by GM-CSF or M-CSF are considered M0 (resting) macrophages [39,53,54,55,56]. GM-CSF-induced M0 macrophages (M1-like; GM-Mac) can be polarized to M1 by Type-II IFN (IFN-γ) plus lipopolysaccharide (LPS) [57,58,59,60,61] or by Th1 cytokine stimulation [57,62]. M1 macrophages produce pro-inflammatory cytokines, including Tumor necrosis factor (TNF)-α, IL-1α, IL-1β, IL-6, and IL-12, as well as chemokines such as C-X-C motif chemokine (CXCL)9, and CXCL10 [63], which initiate the immune responses. M1 macrophages produce nitric oxide (NO) and ROS, and transcription factors, including NF-kB, STAT1, STAT5, IRF3, and IRF5, have been shown to regulate M1 macrophages. Toll-like receptor (TLR)2, TLR4, CD80, CD86, and MHC-II are characteristic surface markers of M1. In contrast, M-CSF-induced M0 macrophages (M2-like/M-Mac) can be polarized into M2a, M2b, M2c, and M2d subtypes [41,43,63]. For example, IL-4 and IL-13 polarize M2-like/M-Mac to M2a [53,56,64,65,66], whereas immune complexes and TLR ligands induce M2b polarization. IL-10, transforming growth factor β (TGF-β), and glucocorticoids promote M2c polarization, and IL-6 or adenosine analogs stimulation can polarize M-Mac toward M2d [41,67,68,69]. These M2 macrophages subsets are regulated by transcription factors including STAT3, STAT6, IRF4, and KLF4 [63,70,71,72], and M2-associated markers include CD80, CD86, CD163, CD206, and CD209 [63,72].

We previously investigated the response of M2-like/M-Mac to IL-27. Since IL-6 polarizes M-Mac toward M2d and IL-27 shares gp130-dependent signaling with IL-6, we hypothesized that IL-27 might polarize M-Mac toward an M2d or M2d-like phenotype. In our prior study, we compared the functions and gene expression profiles of IL-27-polarized M-Mac (27M-Mac) and IL-6-polarized M2d macrophages [27]. We found that 27M-Mac, but not M2d macrophages, were resistant to HIV replication, and only 27M-Mac increased ROS production. Moreover, the gene expression profile of 27M-Mac also differed from that of M2d macrophages. Notably, 27M-Mac expressed CD38 and secreted CXCL9 and C1q. Since CD38 and CXCL9 are associated with M1 macrophages and C1q has been linked to M3 macrophages, we considered that 27M-Mac may resemble GM-CSF-induced M0 (M1-like/GM-Mac), M1-like, M1, or M3. As the effects of IL-27 on GM-Mac remain poorly defined, the current study compares biological functions and transcriptome profiles using single-cell RNA sequencing (scRNA-Seq) analysis among M-Mac, 27M-Mac, GM-Mac, and IL-27-polarized GM-Mac (27GM-Mac) and defines the distinguishing features of 27M-Mac and 27GM-Mac.

## 2. Materials and Methods

### 2.1. Cells and Reagents

Peripheral blood mononuclear cells (PBMCs) were isolated from healthy donors’ apheresis packs obtained from the National Institutes of Health Blood Bank (Bethesda, MD, USA) or STEMCELL (Cambridge, MA, USA) using lymphocyte separation medium (ICN Biomedical, Aurora, OH, USA), as previously described [27]. Monocytes were isolated from PBMCs using CD14 MicroBeads (Miltenyi Biotec, Auburn, CA, USA) according to the manufacturer’s instructions. Cell purity was ≥90%, as determined by flow cytometry. Cell viability was assessed by trypan blue (Thermo Fisher Scientific, Waltham, MA, USA) exclusion. CD14^+^ monocytes were differentiated into M-Mac and GM-Mac macrophages using M-CSF (R&D Systems, Minneapolis, MN, USA) and GM-CSF (R&D Systems), respectively, as previously described [27]. After differentiation, M-Mac and GM-Mac cells were polarized to 27M-Mac and 27GM-Mac by culturing for 3 days in the presence of 100 ng/mL IL-27 (R&D Systems) in complete DMEM (Thermo Fisher Scientific) supplemented with 10% (*v*/*v*) FBS (R&D Systems), 10 mM HEPES, and 10 μg/mL gentamicin (D10 medium), as previously described [10,12,27]. HEK293T cells were obtained from ATCC (Manassas, VA, USA) and maintained in D10 medium. A plasmid encoding HIV-1_AD8_ was obtained from Dr. M. Martin (NIAID, Bethesda, MD, USA) [73]. The plasmid was propagated in STBL3 cells (Thermo Fisher Scientific) and purified using the EndoFree Plasmid Maxi Kit (Qiagen, Germantown, MD, USA) [4]. Infectious HIV-1_AD8_ virus stock was prepared and titrated as described before [4].

### 2.2. HIV-1 Replication Assay

HIV-1 replication assays were performed in 96-well plates as previously described [27]. Macrophages were seeded at 50 × 10^3^ cells/well and cultured overnight at 37 °C in D10 medium, followed by polarization with IL-27 for 3 days as described above. Polarized cells were washed with D10 medium and infected with 250 TCID_50_ HIV-1_AD8_ (multiplicity of infection, MOI = 0.005) for 2 h at 37 °C [5]. Infected cells were cultured for 14 days, with half-medium changes every 3 to 4 days using prewarmed D10 medium. All replication assays were performed in quadruplicate. HIV-1 replication was quantified by measuring p24 antigen in culture supernatants using a p24 antigen capture assay kit (PerkinElmer, Boston, MA, USA).

### 2.3. Quantitation of C1q, and CXCL9 in Culture Supernatants

To compare C1q and CXCL9 production during IL-27 treatment, M-Mac or GM-Mac from four independent donors were cultured for 3 days in the absence or presence of 100 ng/mL of IL-27. Cell-free culture supernatants were collected and stored at −20 °C until analysis. C1q and CXCL9 concentrations were quantified using a Human C1q ELISA kit (Invitrogen, Thermo Fisher Scientific, Waltham, MA, USA; detection limit, 937 pg/mL) and a CXCL9 kit (R&D Systems; detection limit, 11.4 pg/mL), respectively [27].

### 2.4. Flow Cytometric Analysis

Cellular phenotypes were assessed by single-color staining, with an unstained negative control for each cell type as described before [27]. Compensation controls for each fluorochrome were prepared using compensation beads (Supplementary Table S1), and compensation was calculated before acquisition. A total of 3 × 10^6^ cells were washed three times with ice-cold Dulbecco’s PBS (Thermo Fisher Scientific) containing 2% bovine serum albumin (BSA; Sigma-Aldrich, St. Louis, MO, USA) and 0.5% NaN_3_ (MilliporeSigma, Burlington, MA, USA) (DPBS-BSA-NaN_3_). Cells were blocked with Fc Receptor Blocker (Innovex Biosciences, Richmond, CA, USA) for 30 min at room temperature in the dark, washed twice with DPBS-BSA-NaN_3_, and stained with individual antibodies for 15 min at room temperature in the dark, with an unstained negative control. Antibodies are listed in Supplementary Table S1. Cells were washed twice with DPBS-BSA-NaN_3_ and analyzed immediately on an LSRFortessa flow cytometer (BD Biosciences, San Jose, CA, USA). Data were analyzed using FCS Express version 7 (De Novo Software, Pasadena, CA, USA) [74].

### 2.5. Phagocytosis Assay

Endogenous phagocytic activity was measured using *Escherichia coli* (*E. coli*) (K-12 strain) conjugated with pH-sensitive pHrodo BioParticles (Thermo Fisher Scientific). Phagocytosis was assessed by quantifying bacterial internalization; bactericidal (killing/neutralization) activity was not evaluated in this study. BioParticles (2 mg) were reconstituted in 100 μL PBS (Quality Biological Inc, QBI, Gaithersburg, MD, USA) and vortexed for 15 s three times. After confirming a homogeneous suspension, particles were opsonized using the BioParticles Opsonizing Reagent (Thermo Fisher Scientific). Briefly, 30 μL of reconstituted opsonizing reagent was mixed with 30 μL of reconstituted BioParticles (20 mg/mL), vortexed, and incubated at 37 °C for 1 h. The mixture was centrifuged at 1000× *g* for 5 min and washed three times with PBS. Pellets were resuspended in 100 μL PBS, and particle numbers were determined by microscopy. Phagocytosis assays were performed in 96-well plates. M-Mac or GM-Mac cells were polarized in 96-well plates as described above, washed three times with prewarmed D10 medium, and incubated with opsonized fluorescent *E. coli* at a target-to-effect ratio of 10 for 1 h in D10 medium. As a control, cells were treated with 5 μg/mL cytochalasin D (Sigma-Aldrich) for 30 min before the addition of opsonized *E. coli*. Heat-inactivated *E. coli* particles were also included as controls. Images were acquired using an Axio Observer A1 motorized microscope (Zeiss, Oberkochen, Germany). The unopsonized *E. coli* particles were used for the CD14/TLR4-mediated phagocytosis assay under the culture condition described above. Phagocytic activity was quantified using Fiji Version 2.16 (National Institutes of Health, Bethesda, MD, USA). Background thresholds were applied to generate 8-bit masks. For the red channel, the total stained fluorescence area was quantified as a measure of phagocytic staining, and particle counts were obtained following measurement of the staining area. Results are presented as the average fluorescence area per cell for each condition, quantified from images containing approximately 1000 cells.

### 2.6. Autophagy Assay

Autophagy was assessed using the Cyto-ID Autophagy Detection Kit (ENZ-51031; Enzo Life Sciences, Farmingdale, NY, USA) with modifications to a previous report [28]. Polarized cells were incubated overnight at 37 °C in D10 medium with or without 10 μM chloroquine (Enzo Life Sciences). Autophagosomes were stained with the Cyto-ID reagent, and Hoechst 33342 was used as a nuclear counterstain, according to the manufacturer’s instructions. Briefly, cells were washed twice, and culture medium was replaced with PBS supplemented with 5% FBS and containing both stains. Cells were incubated at 37 °C for 30 min in the dark and then imaged using a Zeiss Axio Observer A1 motorized microscope with a 10× objective. Excitation/emission wavelengths were 463/534 nm for the Cyto-ID dye and 350/461 nm for Hoechst 33342. Image analysis was performed using Fiji. Background thresholds were applied to generate 8-bit masks. Total green area was used as a measure of autophagosome staining, and cell numbers were determined by particle counting after watershed segmentation of the nuclear channel. Results are presented as the average stained area per cell for each condition [28].

### 2.7. Reactive Oxygen Species

ROS were quantified using the Amplex Red Hydrogen Peroxide/Peroxidase Assay Kit (Thermo Fisher Scientific), as previously described [5,27]. Cells were stimulated with 100 nM phorbol 12-myristate 13-acetate (PMA) (Sigma-Aldrich) for 30 min at 37 °C, and ROS generation was measured using a Spark microplate reader (Tecan, Männedorf, Switzerland).

### 2.8. Western Blotting

Western blotting (WB) was performed as previously described [5,27]. Briefly, unpolarized or polarized macrophages in 6-well plates (1.5 × 10^6^ cells/well) were washed with cold PBS, lysed using 150 μL 1× Radioimmunoprecipitation assay (RIPA) lysis buffer (Boston BioProducts, Milford, MA, USA) supplemented with 5 mM EDTA (Thermo Fisher Scientific) and 1× phosphatase and protease inhibitor cocktail (Thermo Fisher Scientific). Lysates were incubated on ice for 15 min, and debris-free lysates were obtained by centrifugation at 15,000× *g* at 4 °C for 10 min. Protein concentration was determined using the BCA Protein Assay Kit (Pierce, Thermo Fisher Scientific). For each sample, 20 μg total protein was resolved by SDS-PAGE on NuPAGE Bis-Tris gels (4% to 12%; Thermo Fisher Scientific) in MOPS buffer (Thermo Fisher Scientific) under reducing conditions. Proteins were transferred onto 0.45 μm nitrocellulose membranes (Thermo Fisher Scientific) and probed with antibodies (Supplementary Table S1). Bands were detected using ECL Prime Western Blotting Detection Reagent (Cytiva Life Sciences, Marlborough, MA, USA) and imaged with an Azure 300 system (Azure Biosystems, Dublin, CA, USA). Band intensities were quantified using Fiji.

### 2.9. Construction of scRNA-Seq Libraries

M-Mac or GM-Mac macrophages (4 × 10^6^ cells) were seeded in 60 mm Petri dishes and cultured at 37 °C for ≥16 h in D10 medium. Cells were polarized with 100 ng/mL IL-27 for 3 days. After polarization, cells were washed three times with warm PBS and incubated with 2.5 mL 0.25% trypsin-EDTA (QBI) at 37 °C for 15 min in a 5% CO_2_ incubator. D10 medium (5 mL) was added to stop trypsinization, and cells were detached by gentle flushing using pipets. Detached cells were washed three times with D10 medium, and cell viability and counts were determined using a Cellometer Auto 2000 (Nexcelom Bioscience, Lawrence, MA, USA) with ViaStain AOPI Staining Solution (Nexcelom Bioscience). Cell viability was 98% to 100%. Cells were resuspended at 1 × 10^6^ cells/mL in D10 medium. scRNA-Seq libraries were generated using the Chromium Next GEM Single Cell 5′ Reagent Kit v2 (Dual Index) (10x Genomics, Pleasanton, CA, USA) according to the manufacturer’s instructions. Briefly, 16.5 μL of cell suspension was combined with reverse transcription reagents, template-switch oligonucleotides, and reverse transcriptase, and loaded onto a Chromium Chip K (10x Genomics). Single-cell GEM generation and barcoding, followed by cDNA synthesis, were performed using the 10x Chromium Controller. cDNA was amplified for 13 cycles. Based on Agilent 2100 Bioanalyzer (Agilent Biotechnology, Santa Clara, CA, USA) and Qubit 4 (Thermo Fisher Scientific) analyses, libraries showed a single DNA peak between 300 and 1000 bp (average fragment size, 450 to 550 bp) [27].

### 2.10. Analysis of scRNA-Seq

Sequence data were processed using Cell Ranger v6.0.1 (10x Genomics) as previously described [27]. Resulting count matrices were generated using the standard pipeline with default parameters and imported into Partek Flow (version 12.9.1: https://www.illumina.com/products/by-type/informatics-products/partek-flow.html, accessed on 23 July 2025) for quantification and statistical analysis. Data underwent quality control (QC) and filtering using the following criteria: (1) low-quality cells and potential doublets were removed based on total reads per cell (1000 to 60,000), expressed genes per cell (1000 to 7000), and mitochondrial reads (<15%); and (2) genes with maximum values ≤1 were excluded. Counts were normalized to counts per million, offset by 1, and log_2_-transformed. The top 20 principal components (PCA) were used for unbiased graph-based clustering. Clusters were visualized using t-distributed stochastic neighbor embedding (t-SNE). Differential gene expression was assessed using an analysis of variance (ANOVA) model. Genes were considered differentially expressed at *p* ≤ 0.05 with an absolute fold change ≥3. To adjust *p* values, Tukey’s Honestly Significant Difference (HSD) is used to compare all possible pairs of group means.

### 2.11. Quantitative RT-PCR (qRT-PCR)

Total cellular RNA was isolated using the RNeasy Kit (Qiagen, Germantown, MD, USA), and cDNA was synthesized using TaqMan reverse transcription reagents (Thermo Fisher Scientific) as described previously [27]. mRNA levels of genes of interest were measured by qRT-PCR on a CFX96 real-time system (Bio-Rad, Hercules, CA, USA) [27]. Relative transcript abundance was calculated using the ΔΔCt method with GAPDH as the reference. Normalized expression values were expressed relative to the mean ΔCt of control samples to obtain fold changes. Gene-specific probes were purchased from Applied Biosystems (Thermo Fisher Scientific) and listed in Supplementary Table S1.

### 2.12. Statistical Analysis

Intergroup comparisons were performed using an unpaired *t*-test, one-way or two-way ANOVA (GraphPad, San Diego, CA, USA). *p*-values <0.05 were considered statistically significant: * *p* < 0.05, ** *p* < 0.01, *** *p* < 0.001, **** *p* < 0.0001; *p* > 0.05 was considered not significant (ns).

## 3. Results

### 3.1. Antiviral Effect

#### 3.1.1. Comparison of Anti-HIV Activity

**Figure 1:**
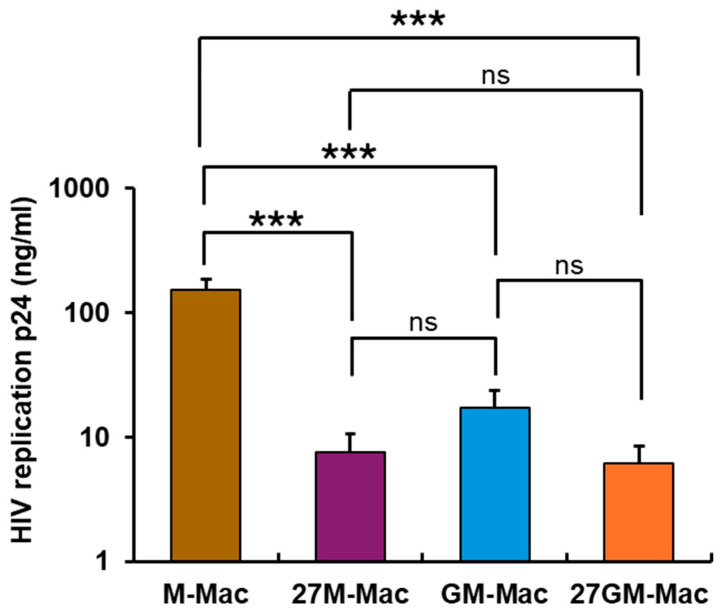
Comparison of HIV replication. M-Mac and GM-Mac were cultured for 3 days in the absence or presence of 100 ng/mL IL-27. The untreated cells (M-Mac and GM-Mac), IL-27–treated/polarized cells (27M-Mac and 27GM-Mac) were infected with HIV_AD8_ at 5000 TCID_50_/1 × 10^6^ cells [multiplicity of infection (MOI) = 0.005] for 2 h at 37 °C. The infected cells were cultured in 200 µL D10 medium for 14 days, with half of the medium changed every 3 to 4 days with fresh warm D10 medium in 96-well plates. All replication assays were conducted in quadruplicate, and HIV-1 replication was determined by measuring the p24 antigen in the culture supernatants using the p24 antigen capture assay kit (PerkinElmer, Boston, MA, USA). Results show mean ± SE from four independent assays. Statistical analysis was conducted using One way ANOVA. ***: *p* < 0.001, ns: not significant.

In our previous study, we investigated the effects of IL-27 on HIV replication and ROS induction in M-Mac. To do so, we treated M-Mac with different dosages of IL-27 for varying incubation periods. Our findings indicated that 100 ng/mL of IL-27 for three days resulted in the most significant effect [5,27]. In order to ascertain whether 100 ng/mL of IL-27 induced the maximum effect on STAT3 activation, different doses of IL-27 were used for 15 min stimulations, and STAT3 activation was assessed. Since 100 ng/mL of IL-27 induced maximum STAT3 activation (Supplementary Figure S1A,B), in the current study, M-Mac and GM-Mac were polarized using 100 ng/mL of IL-27 for a 3-day culture. HIV replication among M-Mac, 27M-Mac, GM-Mac, and 27GM-Mac was compared. Each macrophage subtype was infected with HIV_AD8_ strain at an MOI of 0.005 and cultured for 14 days. HIV replication in culture supernatants was quantified using an HIV-1 p24 antigen capture kit. Consistent with a previous study [27], HIV replication in 27M-Mac was significantly reduced to 4.9 ± 2.0% of that in M-Mac (*n* = 4, *p* < 0.001) (Figure 1). As both M-Mac and GM-Mac are frequently described as M0 or naïve cells [30,75], we speculated that both cell types might exhibit comparable HIV replication. However, HIV replication in GM-Mac was markedly lower than in M-Mac (11.2 ± 4.3% of M-Mac; *n* = 5, *p* < 0.001), which was comparable to that in 27M-Mac (*p* = 0.731). HIV replication in 27GM-Mac was significantly lower than that in M-Mac (4.0 ± 1.4% of M-Mac); however, it was only modestly reduced relative to GM-Mac (36.1 ± 13.5% of GM-Mac; *n* = 5, *p* = 0.166). Thus, IL-27 polarization conferred resistance to HIV replication in both cell types, although the magnitude of the effect differed.

#### 3.1.2. Comparison of Gene Expression Among Four Cell Types

**Figure 2:**
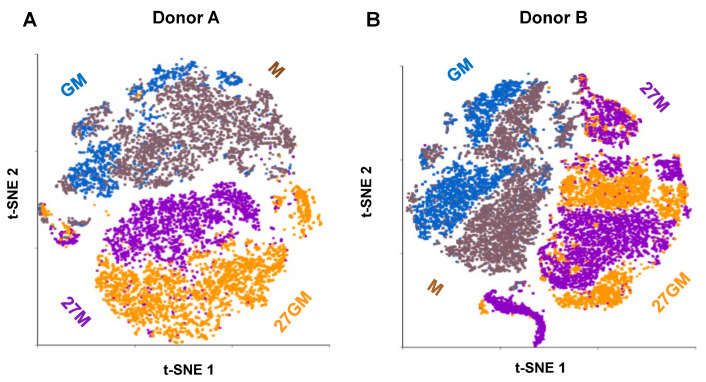
The scRNA-Seq analysis. The scRNA-Seq using fresh M-Mac, 27M-Mac, GM-Mac, and 27GM-Mac cells from two independent donors, Donor A (**A**) and Donor B (**B**), was conducted as described in Section 2. (**A**,**B**) t-distributed Stochastic Neighbor Embedding (t-SNE) plot was used for clustering and visualization. Each dot indicates single cells; M-Mac is brown, 27M-Mac is purple, GM-Mac is blue, and 27GM-Mac is orange.

To characterize the four cell types, we compared their gene expression profiles by single-cell RNA sequencing (scRNA-Seq). Monocytes from two independent donors (Donor A and Donor B) were differentiated and polarized into each cell type and subjected to scRNA-Seq. Quality control yielded 15,619 genes (features) and 11,183 cells from Donor A, and 15,245 genes and 17,427 cells from Donor B. In Donor A, 3676 cells were classified as M-Mac, 2429 as 27M-Mac, 1470 as GM-Mac, and 3608 as 27GM-Mac (Supplementary Table S2). In Donor B, 3808 cells were classified as M-Mac, 5297 as 27M-Mac, 3526 as GM-Mac, and 4796 as 27GM-Mac (Supplementary Table S2). To visualize cell-type distribution and pairwise similarity, we performed t-SNE analysis (Figure 2A,B).

In datasets from both donors, M-Mac clustered closely with GM-Mac, and 27M-Mac clustered closely with 27GM-Mac. IL-27-treated cells formed clusters distinct from untreated M-Mac and GM-Mac. Notably, although 27M-Mac suppressed HIV replication to a similar extent as GM-Mac, the gene expression profile of 27M-Mac was distinct from that of GM-Mac. To define similarities and differences in gene expression, we analyzed differentially expressed genes (DEGs) among the four cell types. As shown in Figure 2A,B, gene expression profiles differed between M-Mac and GM-Mac in both donors. Therefore, comparing DEGs between 27M-Mac and M-Mac (27M vs. M) with those between 27GM-Mac and GM-Mac (27GM vs. GM) could be confounded by baseline differences between M-Mac and GM-Mac, potentially introducing bias. To mitigate this issue, we compared normalized reads for each gene in 27M-Mac, GM-Mac, and 27GM-Mac with the corresponding reads in M-Mac. DEGs were defined with *p* < 0.05 and an absolute fold change ≥3. In Donor A, the numbers of DEGs for 27M vs. M, GM vs. M, and 27GM vs. M were 560, 119, and 398, respectively (Table 1).

In Donor B, the numbers of DEGs for 27M vs. M, GM vs. M, and 27GM vs. M were 406, 52, and 464, respectively (Table 1). The relatively small number of DEGs in GM vs. M suggests that GM-Mac and M-Mac were transcriptionally more similar than the other pairwise comparisons. Additional comparison analysis of 27GM-Mac and 27M-Mac was conducted. The number of DEGs between 27GM-Mac and 27M-Mac (27GM vs. 27M) in Donor A and in Donor B were 219 and 30, respectively (Table 1). These values were lower than those observed in DEGs for 27M vs. M, supporting substantial transcriptional similarity between 27M-Mac and 27GM-Mac and suggesting that IL-27 polarization of M-Mac yields a state comparable to 27GM-Mac.

#### 3.1.3. Potential Mechanisms Underlying Anti-HIV Activity

**Figure 3:**
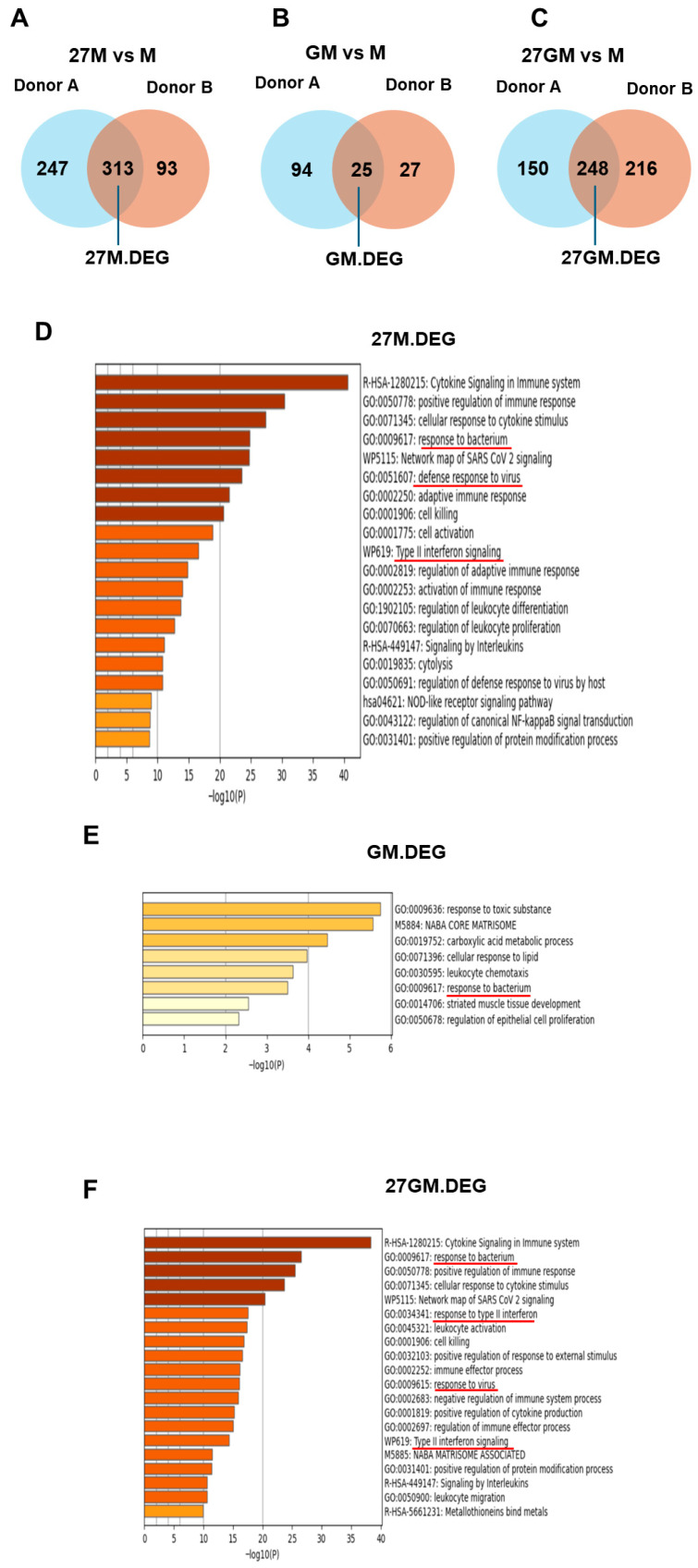
Extraction of common DEGs compared to M-Mac cells and functional enrichment analyses. (**A**–**C**) To identify the common DEGs between donor (**A**) and donor (**B**) for each cell type, gene expression profiles of 27M-Mac, GM-Mac, and 27GM-Mac were compared to those in M-Mac within each donor. Differential expression was defined as genes exhibiting a greater than 3–fold change up- or down-regulation with a *p*-value less than 0.05. DEGs identified from comparisons of 27M-Mac vs. M-Mac (27M vs. M), GM-Mac vs. M-Mac (GM vs. M), and 27GM-Mac (27GM vs. M) were then intersected between donors to define common DEGs using Venn diagrams. (**D**–**F**) The functional enrichment analyses of the common DEGs were conducted using Metascape Version 3.5 (https://metascape.org/, accessed on 18 November 2025) for 27M.DEGs (**D**). GM.DEGs (**E**), and 27GM.DEGs (**F**). Red lines indicate annotation of response to bacterium, defense response to virus, and Type II interferon signaling.

HIV replication in M-Mac was significantly higher than that in 27M-Mac, GM-Mac, and 27GM-Mac, suggesting that the expression of host dependency factor(s) might be suppressed in those cell types compared with M-Mac or that the expression of host restriction factors might be induced in those cell types. To identify the genes commonly associated with anti-HIV activity in 27M-Mac, GM-Mac, and 27GM-Mac across both donors, we first employed Venn diagrams to define common DEGs (combining upregulated and downregulated genes) shared by both donors. This analysis identified 313, 25, and 248 DEGs in common 27M.DEG, GM.DEG, and 27GM.DEG sets, respectively (Figure 3A–C; all DEG names are listed in Supplementary Table S3). We then performed functional annotation of these DEG sets to define biological functions and pathways associated with IL-27 treatment (Figure 3D–F).

**Figure 4:**
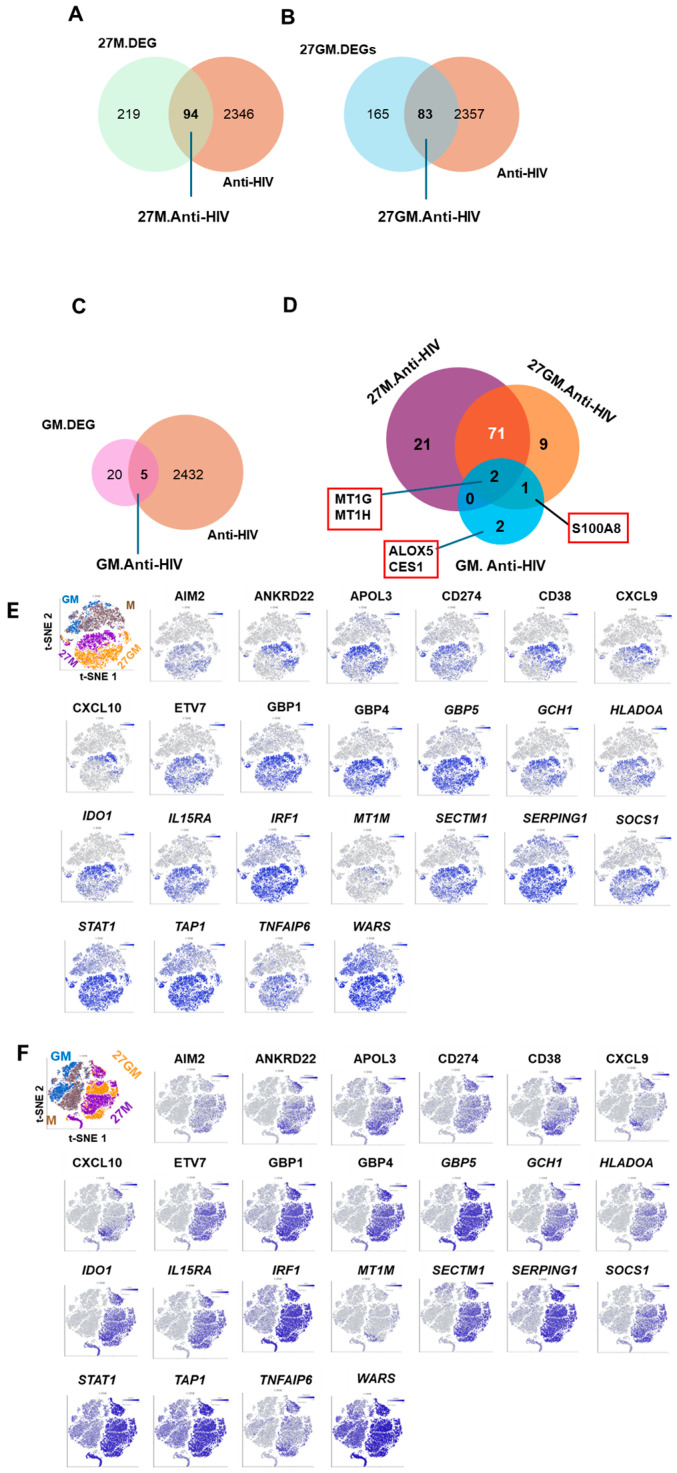

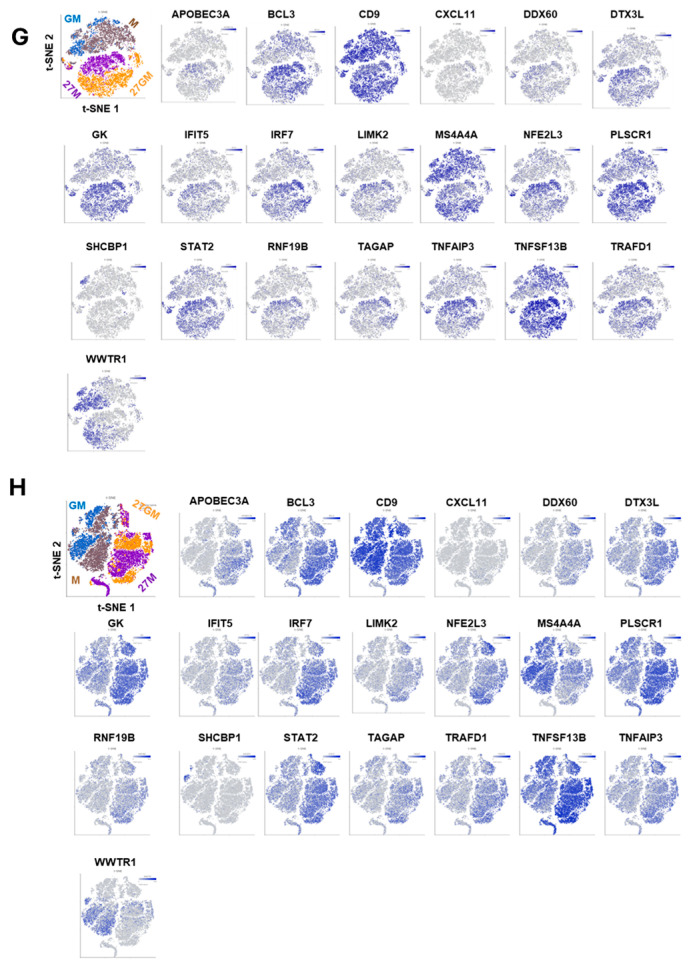
Identification of genes associated with anti-HIV effect. (**A**–**C**) To identify genes associated with the suppression of HIV-1 replication in 27M-Mac ((**A**), 27M.anti-HIV), 27GM-Mac ((**B**), 27GM.Anti-HIV), and GM-Mac ((**C**), GM.Anti-HIV), Venn diagram analyses were conducted between the anti-HIV/HF gene list and corresponding DEGs: 27M.DEGs (Figure 3A), 27GM.DEGs (Figure 3B), and GM.GEGs (Figure 3C). (**D**) To identify common anti-HIV genes shared among 27M-Mac, 27GM-Mac and GM-Mac, a Venn diagram was carried out using the gene lists from 27M.Anti-HIV, 27GM.Anti-HIV and GM.Anti-HIV. The names of genes are listed in a red box alongside the Venn diagram. The names of 71 and 21genes are listed in Supplementary Table S6. (**E**,**F**) t-SNE plots showing expression of the top 24 genes from the common 71 genes in donor A (**E**) and donor B (**F**). (**G**,**H**) t-SNE plots showing the 21-genes in Donor A (**G**) and Donor B (**H**). Blue dots indicate cells expressing the indicated genes, while gray dots represent cells lacking the expression of the gene. The color intensity indicates relative gene expression levels, with gray indicating low or absent expression and darker blue indicating higher expression as shown in color scales.

Comparison of the top five annotations in the common 27.DEGs (Figure 3D) and the common 27GM.DEGs (Figure 3F) showed strong similarity, including genes related to response to bacterium (with red under lines in Figure 3D–F), although the ordering differed. WB demonstrated persistent activation of STAT1 and STAT3 in 27M-Mac and 27GM-Mac over 3 days of culture (Supplementary Figure S1C). As IL-27–mediated STAT1 and STAT3 activation is similar to Type-II IFN (IFN-γ) stimulation [3,6,27] consistently, responses to type II IFNs were annotated in 27M.DEG (Figure 3D, with red underline), 27GM.DEG (Figure 3F, with red under line), and DEGs from 27GM vs. GM (Supplementary Figure S2A). Responses to type I IFN (IFN-α or IFN-β) were annotated only in the DEG set from 27GM vs. GM and were not present in the annotation of 27GM vs. 27M (Supplementary Figure S2B). To identify genes potentially contributing to antiviral activity, we intersected each common DEG set with anti-HIV/host factor genes (Supplementary Table S4) [27]. This analysis identified 94, 83, and 5 antiviral/host factor genes in 27M.DEG (Figure 4A), 27GM.DEG (Figure 4B), and GM.DEG (Figure 4C), respectively (gene lists are provided in Supplementary Table S5).

As 27M-Mac, GM-Mac, and 27GM-Mac demonstrated nearly 10% of HIV replication compared to M-Mac, to determine whether the shared antiviral/host factor genes among the three cell types existed, we performed a Venn diagram analysis using these three gene lists (Figure 4D). Two genes, *MT1G* and *MT1H*, were common among all three cell types; 71 genes (listed in Supplementary Table S6) overlapped between 27M.Anti-HIV and 27GM.Anti-HIV, and 21 genes (listed in Supplementary Table S6) were DEGs in 27M.Anti-HIV. To visualize the distribution of antiviral gene expression, we performed t-SNE analysis focusing on the top 24 genes showing >10-fold differences relative to M-Mac (Figure 4E,F). Some antiviral genes (for example, *GBP1*, *GBP4*, *GBP5*, *TAP1*, and *SERPING1*) were expressed in nearly all 27M-Mac and 27GM-Mac cells, whereas others (for example, *ANKRD22*, *APOL3*, *CXCL9*, *CXCL10*, and *MT1M*) were restricted to subpopulations of 27M-Mac and 27GM-Mac. The same t-SNE analysis was employed for the 21 unique genes in 27M. Anti-HIV (Figure 4G,H and Supplementary Table S7). The expression levels of individual genes and the population of cells expressing each gene varied among cells (Supplementary Table S8), suggesting that they may contribute to the robust anti-HIV activity of 27M-Mac in concert with the 71 shared genes. Antiviral mechanisms may differ across individual cells and across cell types, as previously described [27].

### 3.2. CD38 Expression and CXCL9 and C1q Production

**Figure 5:**
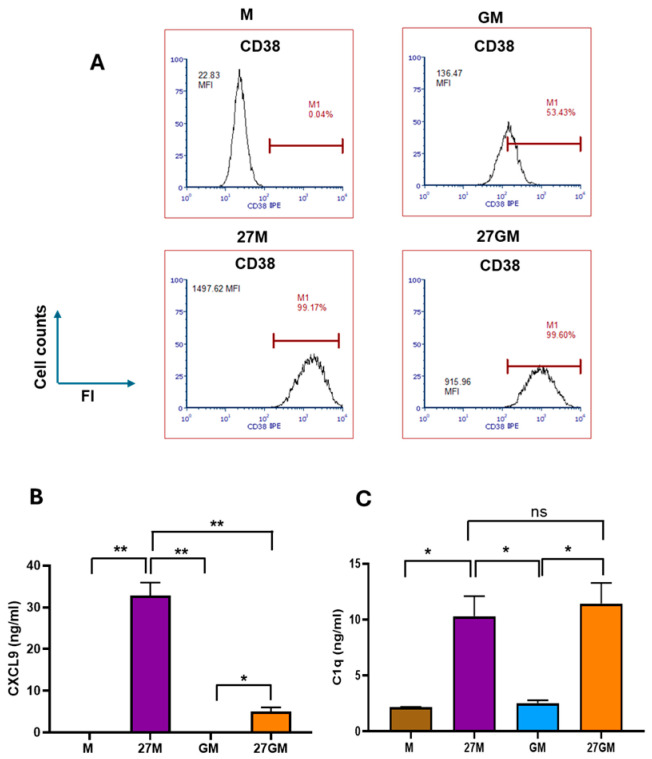
Comparison of CD38 expression and the secretion of CXCL9 and C1q. (**A**) CD38 expressions on the cell surfaces of M-Mac, 27M-Mac, GM-Mac, and 27GM-Mac were analyzed using flow cytometry as described in Section 2. Representative results of CD38 staining from three independent essays are presented. Each isotype control was used to set positive gates shown using horizontal bars on histograms. (**B**,**C**) M-Mac or GM-Mac were cultured for 3 days in the absence or presence of IL-27, and then cell-free supernatants were collected. The levels of secreted CXCL9 (**B**) and C1q (**C**) were determined using an ELISA kit. Results indicate mean ± SE from four independent donors. Statistical analysis was conducted using an unpaired *t*-test. *: *p* < 0.05, **: *p* < 0.01, ns: not significant.

In our previous work, we demonstrated that 27M-Mac expresses CD38 (an M1 marker) and secretes CXCL9 (an M1 marker) and C1q (an M3 marker). Therefore, we compared the expression of CD38 and secretion of CXCL9 and C1q across the four macrophage subtypes. FACS analysis showed that 12 ± 11% (*n* = 3) of M-Mac were CD38^+^, whereas the CD38^+^ population increased to 96 ± 3.0% (*n* = 3) in 27M-Mac (*p* < 0.01). In contrast, 41 ± 15% of naïve GM-Mac were CD38^+^, and the CD38^+^ population increased to 92 ± 6% in 27GM-Mac (*p* < 0.01) (Figure 5A and Supplementary Figure S3). The mean fluorescence intensity (MFI) of CD38 increased by 46.2 ± 6.7-fold in 27M-Mac relative to M-Mac (*n* = 3, *p* < 0.05) and by 5.6 ± 0.9-fold in 27GM-Mac relative to GM-Mac (*n* = 3, *p* < 0.05). Thus, the frequency of CD38-positive cells was comparable between 27M-Mac and 27GM-Mac.

CXCL9 and C1q production over 3 days was quantified in culture supernatants. CXCL9 production was significantly increased in 27M-Mac compared to M-Mac (*p* < 0.01) and in 27GM-Mac relative to GM-Mac (*p* < 0.05). CXCL9 secretion by 27M-Mac was 6.6 ± 3.9-fold higher than that by 27GM-Mac (*p* < 0.01) (Figure 5B). Low levels of C1q were detected in supernatants from M-Mac and GM-Mac, whereas C1q production increased in both 27M-Mac and 27GM-Mac (Figure 5C). The amount of secreted C1q was comparable between 27M-Mac and 27GM-Mac (Figure 5C). Taken together, in terms of cellular marker expression, 27M-Mac and 27GM-Mac exhibited M1- and M3-like characters.

### 3.3. Macrophage Marker Expression

**Figure 6:**
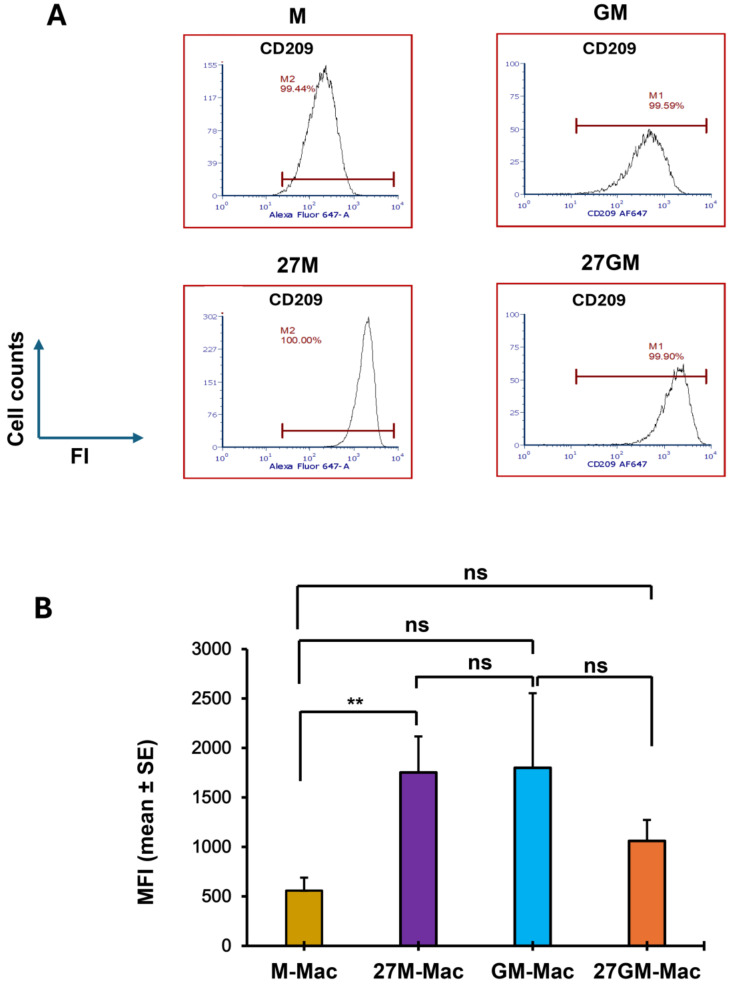
Comparison of the expression of macrophage markers. (**A**) The expression of CD209 on M-Mac, 27M-Mac, GM-Mac, and 27GM-Mac was compared using FACS, as shown in Section 2. An isotype control was used to set positive gates, shown using horizontal bars on histograms. Results are representative of data from three independent experiments. (**B**) MFI calculated from three independent donors was calculated. Data are presented as mean ± SE (*n* = 3). Statistical analysis was conducted using One-way ANOVA. **: *p* < 0.01, ns: not significant.

Expression of macrophage markers (CD80, CD86, CD163, CD206, and CD209) [63] was assessed by flow cytometry in M-Mac, 27M-Mac, GM-Mac and 27GM-Mac from three independent donors. The percentage of cells expressing each marker did not differ among cell types (Supplementary Figure S4), and the MFI of CD80, CD86, CD163, and CD206 showed no significant differences across groups (Supplementary Figure S5A,B). In contrast, the CD209 MFI value was found to be significantly lower in M-Mac than in GM-Mac (*p* < 0.01). However, it increased by 3.13 ± 0.65-fold in 27M-Mac (*p* < 0.01), reaching levels comparable to those of GM-Mac (Figure 6A,B). Of interest, the expression of CD209 was modestly downregulated in 27GM-Mac compared to GM-Mac; however, it was comparable to that in 27M-Mac. Thus, with respect to CD209 expression, 27M-Mac resembled 27GM-Mac.

### 3.4. ROS-Inducing Activity

#### 3.4.1. Comparison of ROS-Inducing Activity

**Figure 7:**
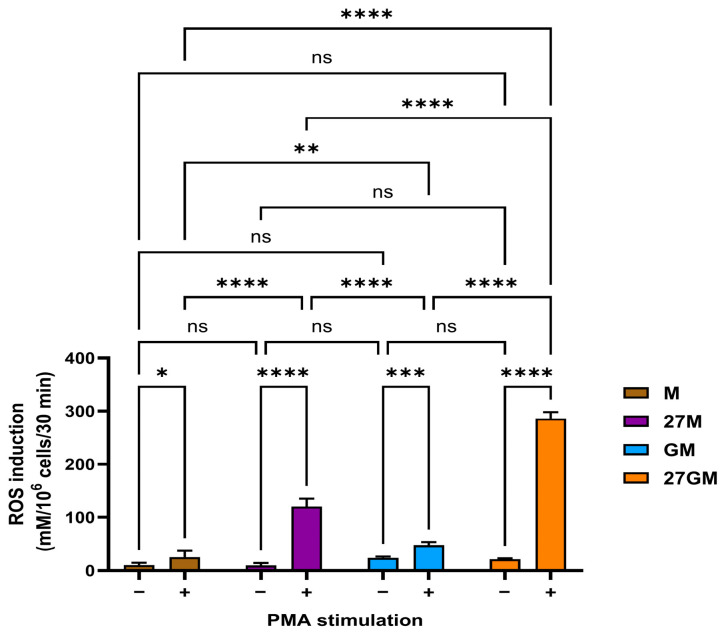
Comparison of ROS inducing activity. M-Mac, 27M-Mac, GM-Mac and 27GM-Mac washed with D-PBS-glucose (PBS-G) and then stimulated without or with 100 nM PMA in PBS-G for 30 min at 37 °C in the presence of Amplex Red Hydrogen Peroxide/Peroxidase Assay Reagent. Induced ROS amounts were determined using the Spark TECAN (Tecan, Männedorf, Switzerland) [27]. All assays were run in quadruplicate in 96-well black plates. Results are representative of three independent experiments and presented as mean ± SD. Statistical analysis was conducted using Two-way ANOVA. *: *p* < 0.05, **: *p* < 0.01, ***: *p* < 0.001, ****: *p* < 0.0001, ns: not significant.

IL-27 treatment enhanced PMA-induced ROS production [5]. Therefore, we compared ROS-inducing activity among the four macrophage subtypes using the same method. Cells were stimulated with PMA, and ROS induction was measured. In the absence of PMA, endogenous ROS levels did not differ significantly between M-Mac and 27M-Mac. In contrast, GM-Mac and 27GM-Mac exhibited modestly higher endogenous ROS levels than M-Mac and 27M-Mac (Figure 7). Following PMA stimulation, ROS induction in 27M-Mac and 27GM-Mac increased significantly by 4.7 ± 0.6-fold (*p* < 0.0001) and 5.6 ± 0.8-fold (*p* < 0.0001), respectively, compared to M-Mac and GM-Mac. ROS production in 27M-Mac was lower than that in 27GM-Mac (*p* < 0.0001).

#### 3.4.2. Mechanisms Underlying Enhanced ROS Production

**Figure 8:**
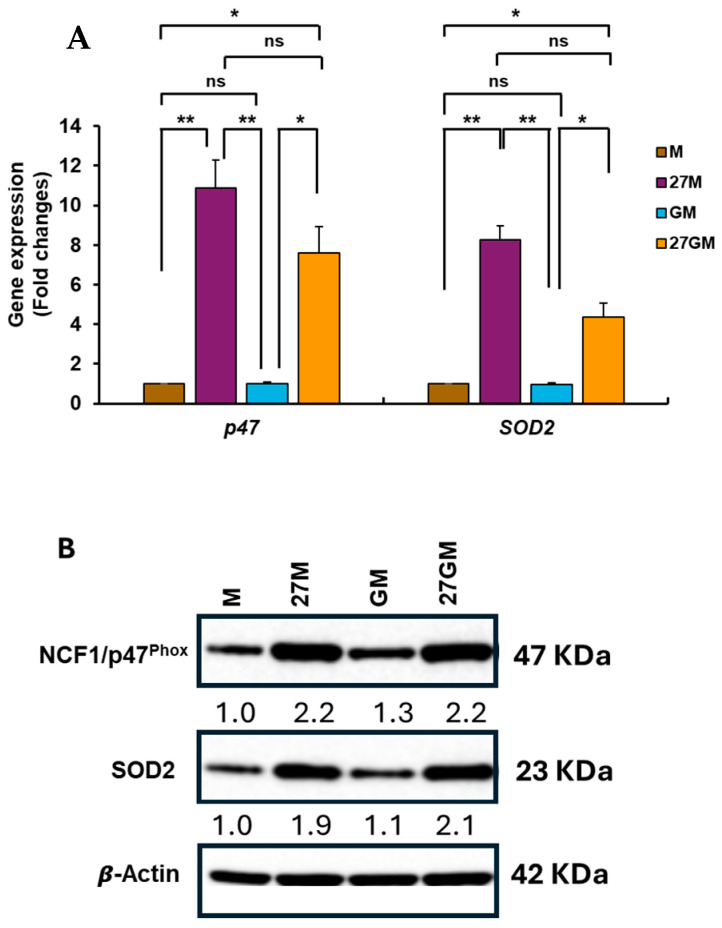
Comparison of gene and protein expression. (**A**) The qRT-PCR was conducted to define the expression level of NCF-1/p47^phox^ and SOD2 using total cellular RNA from M-Mac, 27M-Mac, GM-Mac and 27GM-Mac from four independent donors. Statistical analysis was conducted using one-way ANOVA with Dunnett’s multiple comparisons. Data show mean ± SE, *: *p* < 0.05, **: *p* < 0.01, ns: not significant (**B**) The total cellular protein lysate was collected from M-Mac, 27M-Mac, GM-Mac and 27GM-Mac, and Western blotting was performed using anti-47^Phox^, anti-SOD2, and anti-β-Actin antibodies. The band intensity of each protein was analyzed using Fiji-ImageJ Version 2.16 and then normalized by the band intensity of β-Actin. The resulting values are indicated in the image as fold change compared to the intensity of M-Mac. Results are representative of two independent experiments.

**Figure 9:**
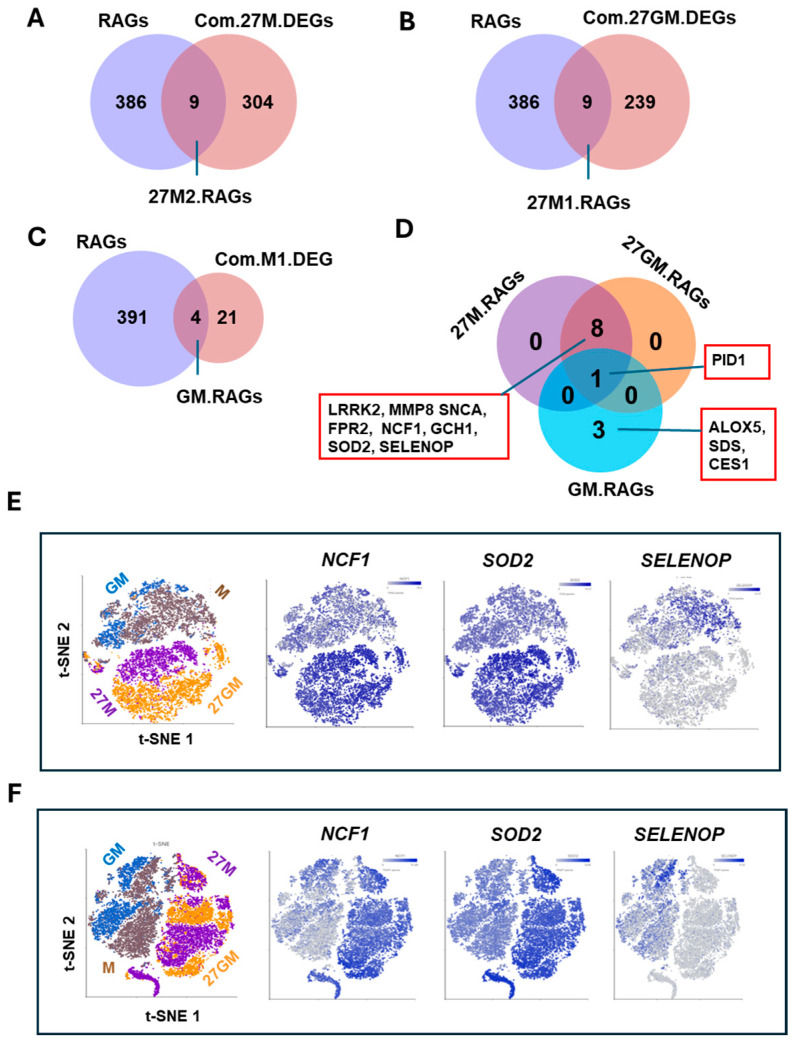
Comparison of the expression of genes associated with ROS. (**A**–**D**) To identify ROS-associated genes (RAGs) in Com.27M.DEGs (**A**), in Com.27GM.DEGs (**B**), and in Com.GM.DEGs (**C**), a Venn diagram analysis was conducted between each DEG and the total RAG gene list. (**D**) To compare similarity among 27M.RAGs, 27GM.RAGs, and GM.RAGs, a Venn diagram analysis was carried out. (**E**,**F**) t-SNE plots demonstrate the distribution of SOD2 and NCF1/P47PHOX gene expression in Donor A (**E**) and Donor B (**F**). Blue dots indicate cells expressing the indicated genes, while gray dots represent cells lacking the expression of the gene. The color intensity indicates relative gene expression levels, with gray indicating low or absent expression and darker blue indicating higher expression.

IL-27 treatment of M-Mac increases expression of neutrophil cytosolic factor 1 (NCF1)/p47^phox^ and superoxide dismutase 2 (SOD2), thereby enhancing the potential for ROS induction [5,27]. We therefore compared NCF1/p47^phox^ and SOD2 expression among the four cell types. qRT-PCR analysis of total cellular RNA illustrated that *NCF1*/*P47PHOX* and *SOD2* were significantly induced in 27M-Mac and 27GM-Mac compared with M-Mac and GM-Mac, respectively (Figure 8A). *NCF1*/*P47PHOX* expression in 27GM-Mac was comparable to that in 27M-Mac, whereas *SOD2* expression in 27GM-Mac was lower than in 27M-Mac (*p* < 0.01). Consistently, WB showed that NCF1/p47^phox^ protein expression increased by a 2.2–fold in both 27M-Mac and 27GM-Mac, whereas SOD2 protein expression increased by 1.9–fold in 27M-Mac and 2.0-fold in 27GM-Mac (Figure 8B). To determine whether the existence of DEGs related to ROS production in 27M-Mac and 27GM-Mac, Venn diagram analysis was performed using 27M.DEGs and27GM.DEGs. Given that the endogenous ROS induction from GM-Mac was merely higher than that from M-Mac, GM.DEGs was also included in the analysis to identify the factor that may be related to endogenous activity (Figure 3A–C). We cross-referenced these DEGs with 395 known ROS-associated genes (RAGs) (Supplementary Table S4) [76,77,78]. Nine genes in 27M.DEGs and nine genes in 27GM.DEGs overlapped with the RAG set, whereas four genes in GM.DEGs overlapped with the RAG set (Figure 9A–C). Additional Venn diagram analysis identified complete overlap between the nine 27M.RAGs and the nine 27GM.RAGs (Figure 9D): *NCF1*/*P47PHOX*, *SOD2*, leucine-rich repeat kinase 2 *(LRRK2*), matrix metalloproteinase 8 *(MMP8*), synuclein alpha (*SNCA*), formyl peptide receptor 2 (*FPR2*), GTP cyclohydrolase 1 (*GCH1*), selenoprotein P (*SELENOP*), and phosphotyrosine interaction domain containing 1 (*PID1*). PID1 was shared among all three cell types, and scRNA-Seq indicated that *PID1* expression in 27M-Mac, 27GM-Mac, and GM-Mac was reduced relative to M-Mac (Supplementary Table S10). PID1 has been reported to exert bidirectional effects on oxidative stress-mediated ROS induction, functioning as either an enhancer or a suppressor (Yang, 2023) [79]. However, PID1 primarily affects mitochondria-mediated ROS production, whereas this study focused on NADPH oxidase-mediated ROS induction on the plasma membrane; therefore, PID1 is unlikely to contribute substantially under our experimental conditions. The remaining eight genes were shared between 27M.RAGs and 27GM.RAGs, suggesting that enhanced ROS induction in 27M-Mac and 27GM-Mac is regulated by similar mechanisms in both cell types. We next evaluated the distribution of *NCF1*/*P47PHOX* and *SOD2* expression using t-SNE plots (Figure 9E). Between 95.0% and 99.5% of 27M-Mac cells and 97.0% to 98.0% of 27GM-Mac cells expressed *NCF1*/*P47PHOX* and *SOD2* (Supplementary Table S11). These data suggested that elevated ROS-inducing potential is driven by NCF1/p47^Phox^ expression, whereas co-expression of SOD2 may limit ROS-mediated cellular damage. SELENOP protein acts as an antioxidant that contributes to cellular redox homeostasis and protection from oxidative damage [77,78]; thus, we considered it as a potential modulator of ROS production. The *t*-SNE analysis indicated that *SELENOP* expression was downregulated in 27M-Mac and 27GM-Mac (Figure 9E,F); therefore, SELENOP is unlikely to directly contribute to enhanced ROS production in these cells.

### 3.5. Phagocytosis Activity

#### 3.5.1. Comparison of Phagocytic Activity

**Figure 10:**
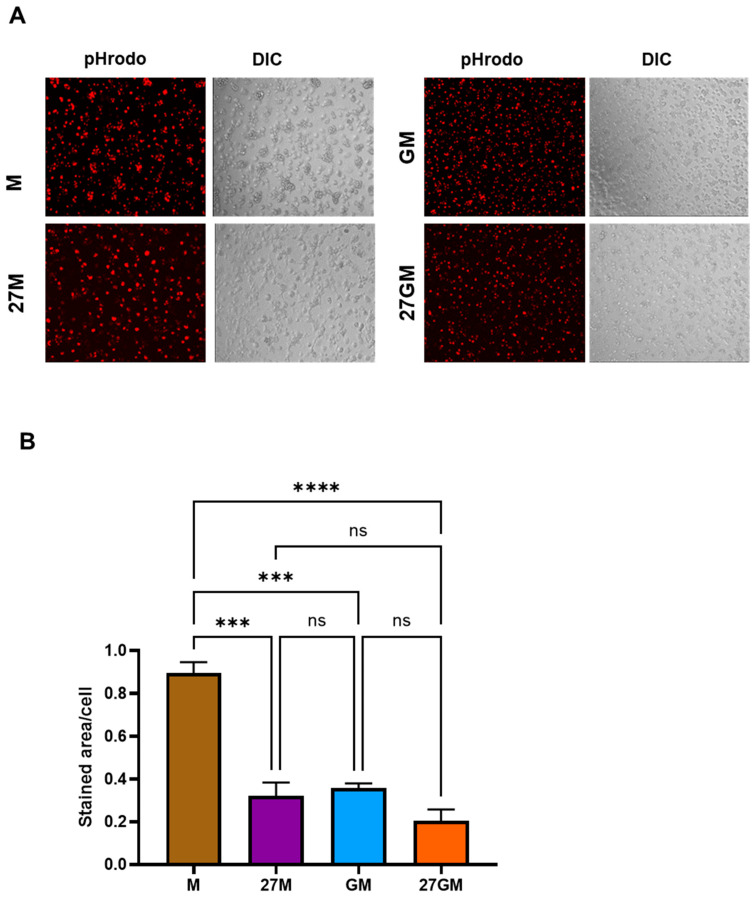

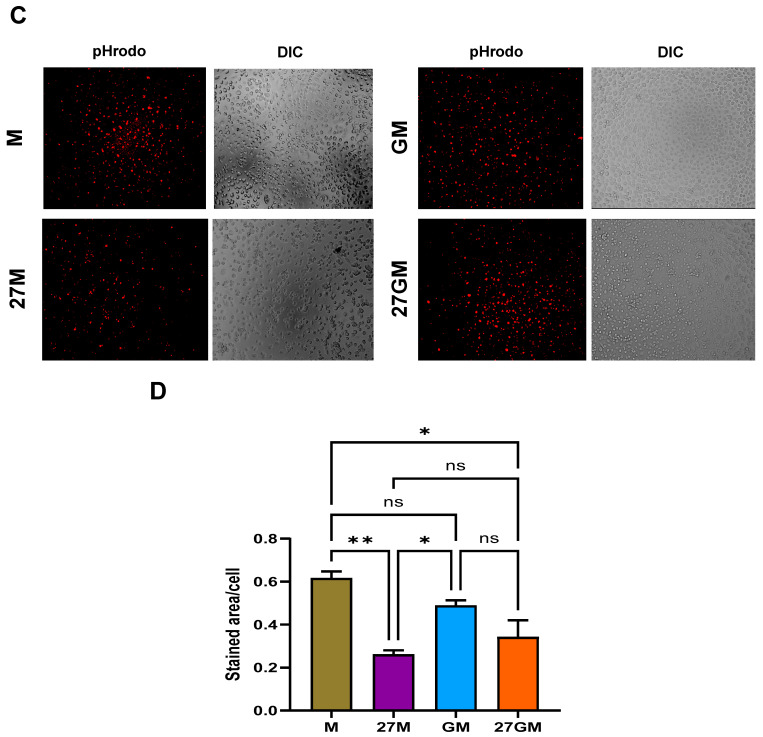
Comparison of phagocytosis activity. M-Mac, 27M-Mac, GM-Mac, and 27GM-Mac were cultured with or without (**A**,**B**) opsonized *E. coli* BioParticles conjugate or (**C**,**D**) un-opsonized BioParticles conjugate (T:E ratio at 10) in 96-well plates for 2 h at 37 °C. The cells were washed, and the uptake of *E. coli*, indicated by red color, was determined using a fluorescence microscope. (**A**,**C**) The phagocytosis images in one of three independent assays are shown. PHrodo Red fluorescence indicates phagocytic cells, while differential Interference Contrast (DIC) images show all cell morphology. (**B**,**D**) Image analysis was performed using the Fiji ImageJ software (Version 2.16). For each channel, a background threshold was set up to create 8-bit masks. For the red channel, the total stained area was retrieved as a measure of phagosome staining, while particle counts following watershed processing of the blue channel gave the corresponding cell number. Results, therefore, display the average stained area per cell for each experimental condition. Data show mean ± SE (*n* = 3) of three independent assays. Statistical analysis was conducted using One-way ANOVA. *: *p* < 0.05, **: *p* < 0.01, ***: *p* < 0.001, ****: *p* < 0.0001, ns: not significant.

To compare phagocytic activity among the four macrophage subtypes, we used a fluorescence microscopy-based assay and quantified phagocytosis as stained area per cell. In this assay, we employed the identical culture condition in conjunction with the anti-HIV and ROS production assay to generate comparative results. This Quantification was performed for 1000 randomly selected cells using ImageJ [28]. All subsets were incubated with opsonized pHrodo BioParticles Red-labeled *E. coli*, and only internalized *E. coli* generated a pHrodo Red signal (Figure 10A). Among the subtypes examined, M-Mac exhibited the highest bacterial internalization (phagocytic uptake) activity (Figure 10B). Phagocytic activity in 27M-Mac was significantly reduced by 64 ± 13% (*n* = 3) compared with M-Mac (*p* < 0.001) and was comparable to that in GM-Mac and 27GM-Mac. Thus, 27M-Mac displayed phagocytic activity similar to 27GM-Mac and GM-Mac. To determine whether the suppression of phagocytosis is specific to opsonized *E. coli* particles, we next assessed CD14–TLR4–mediated phagocytic activity using unopsonized *E. coli.* The uptake of *E. coli* by 27M- and 27GM-Mac was significantly lower than that observed in M-Mac, and the activity in 27M-Mac was comparable to that in 27GM-Mac (Figure 10C,D). Taken together, these results indicated that IL-27-polarized macrophages exhibit reduced uptake of un-opsonized *E. coli* despite increased receptor expression.

#### 3.5.2. Potential Mechanisms Regulating Phagocytosis

**Figure 11:**
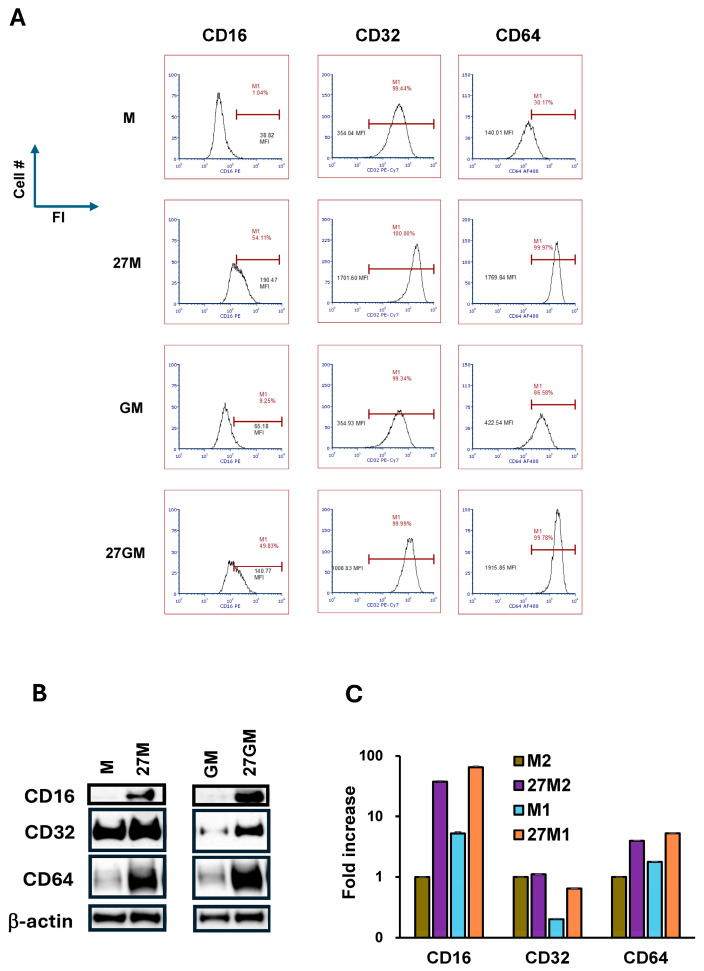
Comparison of FCR expression and the expression of phagocytosis-associated gene. (**A**) Expression of FcγRIII (CD16), FcγRII (CD32), and FcγRI (CD64) on each M-Mac, 27M-Mac, GM-Mac and 27GM-Mac was analyzed by FACS as described in Section 2. Cells from three independent donors were subjected to analysis, and one of the representative data sets is shown in histograms. Each isotype control was used to set positive gates, shown using horizontal bars on histograms. x-axes and Y-axes show fluorescence intensity (FI) and Cell number (Cell # (**B**) The total cellular protein lysate was collected from M-Mac, 27M-Mac, GM-Mac and 27GM-Mac, and WB was performed using anti-CD16, anti-CD32, anti-CD64, and anti-β-Actin antibodies. (**C**) Band intensities were quantified using ImageJ Version 2.16 and normalized by the band intensity of β-Actin.

Since the phagocytosis of opsonized particles in 27M-Mac was markedly lower than in M-Mac (*p* < 0.001) but was comparable to GM-Mac and 27GM-Mac (Figure 10B), we hypothesized that the reduced phagocytosis in 27M-Mac was caused by the decrease in the expression of immunoglobulin G Fc receptors (FcγRs) on the cell surface. We therefore compared FcγR expression across the four subtypes. Flow cytometry showed that each MFI for CD16, CD32, and CD64 increased by approximately 5–10-fold in 27M-Mac and 27GM-Mac (Figure 11A). To determine whether total protein amounts increased, we performed WB on whole-cell lysates and then quantified the amount of each FcγR by normalizing to β-Actin. This analysis demonstrated that total CD16 was 37 ± 1.5-fold higher in 27M-Mac than in M-Mac. In contrast, CD16 abundance in GM-Mac and 27GM-Mac was 5.3 ± 0.37-fold and 64 ± 2.9-fold higher than in M-Mac, respectively (Figure 11B,C). CD32 abundance in 27M-Mac was comparable to that in M-Mac, whereas CD32 in GM-Mac was ~20% of the M-Mac level. CD32 in 27GM-Mac increased by nearly 3-fold relative to GM-Mac (Figure 11C). CD64 abundance increased 4.0 ± 0.06-fold in 27M-Mac compared with M-Mac and increased 1.8 ± 0.02-fold in GM-Mac and 5.3 ± 0.01-fold in 27GM-Mac relative to M-Mac. To examine whether the reduction in uptake was due to the downregulation of CD14 and TLR4 expression, we analyzed their expression levels in 27M- and 27GM-Mac using qRT-PCR, WB, and FACS. qRT-PCR analysis revealed that *CD14* expression was significantly increased by 3.3 ± 0.8-fold (*p* < 0.05, *n* = 3) in 27M-Mac compared with M-Mac, and by 1.7 ± 0.2-fold (*p* < 0.05, *n* = 3) in 27GM-Mac compared with GM-Mac (Supplementary Figure S6A). WB resulted in the total amounts of CD14 were not changed (Supplementary Figure S6B), FACS analyses demonstrated that CD14 expression on the cell surface was increased in 27-M and 27GM-Mac: the MFI for CD14 was increased by 2 to 3-fold (Supplementary Figure S6C). In contrast, qRT-PCR demonstrated that increased TLR4 mRNA expression in both 27M- and 27GM-Mac (3.0 ± 0.5-fold and 1.6 ± 0.2-fold increase, respectively; *p* < 0.05, *n* = 3; compared with M-Mac and GM-Mac, respectively; Figure S6A). This is in line with the report that IL-27 enhances TLR4 expression in monocytes [80]. However, WB demonstrated that the total amount of TLR4 protein was slightly changed in 27-Mac and 27GM-Mac, and FACS analyses illustrated that TLR4 expression was modestly increased in 27M-Mac or 27GM-Mac but not downregulated (Supplementary Figure S6B,C). Taken together, reduced phagocytosis/uptake was unlikely associated with the expression of FcγR, CD14, or TLR4 levels. Instead, suppression may reflect intracellular regulations. Since residual phagocytic activity in 27M-Mac resembled that in GM-Mac and 27GM-Mac, we reasoned that 27M-Mac might upregulate genes encoding phagocytosis inhibitors and/or downregulate genes encoding phagocytosis inducers relative to M-Mac, with similar changes occurring in 27GM-Mac.

**Figure 12:**
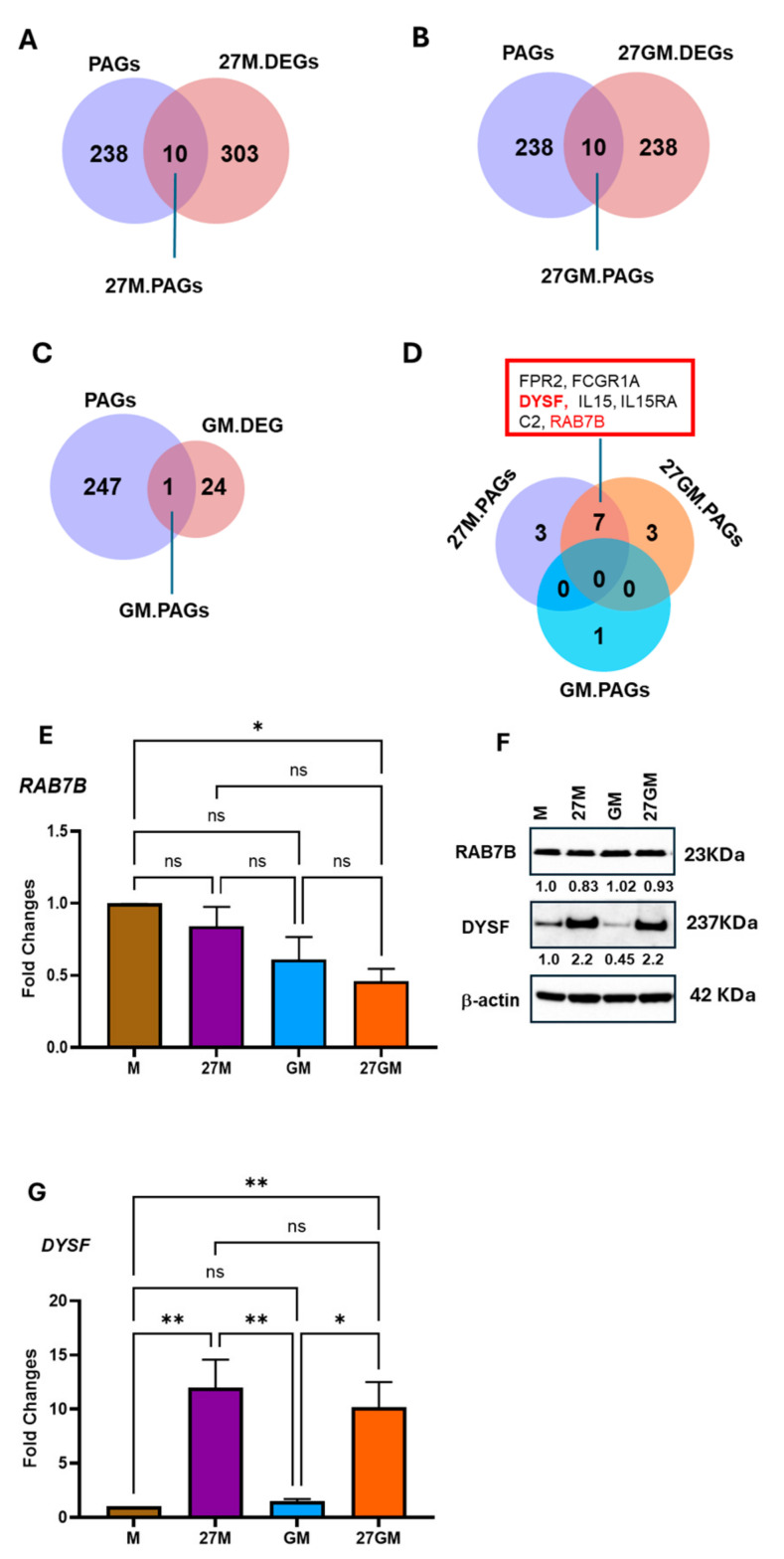

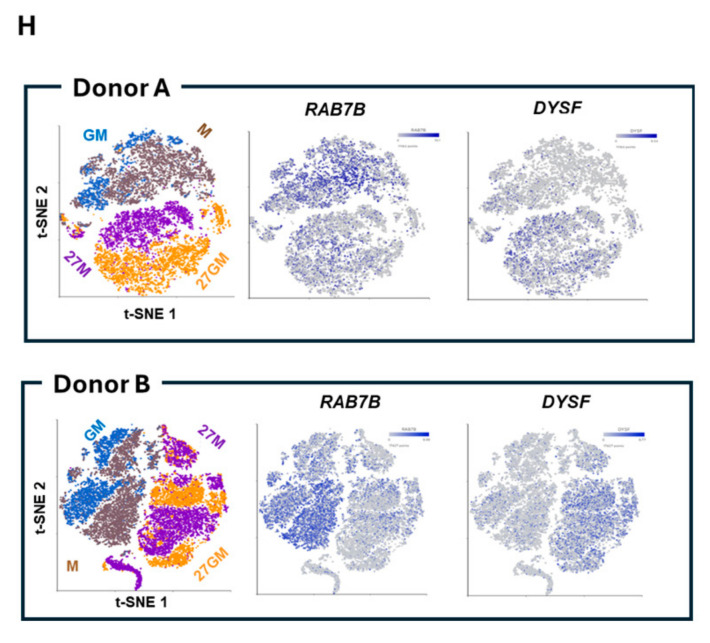
Comparison of the expression of phagocytosis-associated genes. (**A**–**C**) To identify phagocytosis-associated genes (PAGs) within each DEG, Venn diagram analyses were performed between PAGs and 27M.DEGs (**A**), between PAGs and 27GM.DEGs (**B**), and between PAGs and GM.DEGs (**C**). (**D**) To define common PAGs among cell types, a Venn diagram was conducted using 27M.PAGs, 27GM.DEGs and GM.DEGs. (**E**,**F**) The qRT-PCR was performed to define the expression level of RAB7B (**E**) and DYSF (**F**) using total cellular RNA from M-Mac, 27M-Mac, GM-Mac and 27GM-Mac from four independent donors. Statistical analysis was conducted using one-way ANOVA with Dunnett’s multiple comparisons test. Data show mean ± SE. *: *p* < 0.05, **: *p* < 0.01, ns: not significant. (**G**) The total cellular protein lysate was collected from M-Mac, 27M-Mac, GM-Mac and 27GM-Mac, and WB was performed using anti-RAB7B, anti-DYSF, and anti-β-Actin antibodies. The band intensity of each protein was normalized by the band intensity of β-Actin using ImageJ (version 12.6) The resulting values are indicated in the image as fold change compared to the intensity of M-Mac. (**H**) t-SNE plots showing the distribution of RAB7B and DYSF expression across cell populations from donors A and B. Blue dots indicate cells expressing the indicated genes, while gray dots represent cells lacking the expression of the gene. The color intensity indicates relative gene expression levels, with gray indicating low or absent expression and darker blue indicating higher expression as shown in color bars.

To identify potential regulators, we intersected each of the three DEG sets (27M.DEG, 27GM.DEG, and GM.DEG; Figure 12A–D) with 248 phagocytosis-associated genes (PAGs) curated from available phagocytosis databases (Table S4: https://maayanlab.cloud/Harmonizome/gene_set/phagocytosis/GO+Biological+Process+Annotations+2023, accessed on 23 July 2025; https://www.gsea-msigdb.org/gsea/msigdb/cards/%20GOBP_PHAGOCYTOSIS, accessed on 23 July 2025). Ten genes in 27M.DEG (Figure 12A), ten genes in 27GM.DEG (Figure 12B), and one gene in GM.DEG (Figure 12C) overlapped with the PAG list as 27M.PAGs, 27GM, and PAGs and GM.PAG, respectively. To assess similarity between 27M-Mac and 27GM-Mac, we performed an additional Venn analysis, which identified seven overlapping PAGs between 27M.PAG and 27GM.PAG (Figure 12D). Among these seven genes, Ras-related protein B (*RAB7B*) and dysferlin (*DYSF*) encode intracellular proteins, whereas the remaining genes encode receptors or soluble factors (showed in bold font in the red box of Figure 12D). We therefore prioritized *RAB7B* and *DYSF* as candidate intracellular regulators of phagocytosis. *DYSF* is a negative regulator of phagocytosis, whereas *RAB7B* contributes to intracellular trafficking in macrophages [81]. qRT-PCR analysis in three independent donors confirmed expression patterns for these genes. *RAB7B* expression in 27M-Mac and GM-Mac was modestly reduced compared with M-Mac (Figure 12E). Although *RAB7B* expression in 27GM-Mac was significantly lower than that in M-Mac (*p* < 0.05), WB demonstrated that amounts of RAB7B protein expression did not significantly differ among the four subtypes (Figure 12F). In contrast, expression of the negative regulator *DYSF* was significantly increased in 27M-Mac and 27GM-Mac compared with M-Mac (*n* = 3, *p* < 0.01) and GM-Mac (*n* = 3, *p* < 0.05), respectively (Figure 12G). WB confirmed a 2.2 ± 0.02-fold increase in DYSF protein abundance in 27M-Mac relative to M-Mac (Figure 12F). Of interest, DYSF protein expression in GM-Mac was 57 ± 2% of M-Mac, while the protein expression in 27GM-Mac was comparable to 27M-Mac (DYSF in 27GM-Mac was 2.2 ± 0.06-fold higher than that in M-Mac), suggesting that the increased DYSF may contribute to phagocytic suppression in 27M-Mac and 27GM-Mac. Despite reduced phagocytic activity in GM-Mac, DYSF protein expression was suppressed; SLENOP may not play a key role in the inhibition of phagocytosis in this cell type. We next examined the distribution of *RAB7B*- and *DYSF*-expressing cells using t-SNE plots (Figure 12H and Supplementary Table S12). The fraction of cells expressing *DYSF* increased by~4–5-fold in 27M-Mac and 27GM-Mac relative to M-Mac and GM-Mac, whereas *RAB7B* expression in 27M-Mac and 27GM-Mac decreased by ~30–50% relative to M-Mac (Supplementary Table S12).

### 3.6. Autophagy Promotion

#### 3.6.1. Comparison of Promotion of Autophagic Vesicle Formation

**Figure 13:**
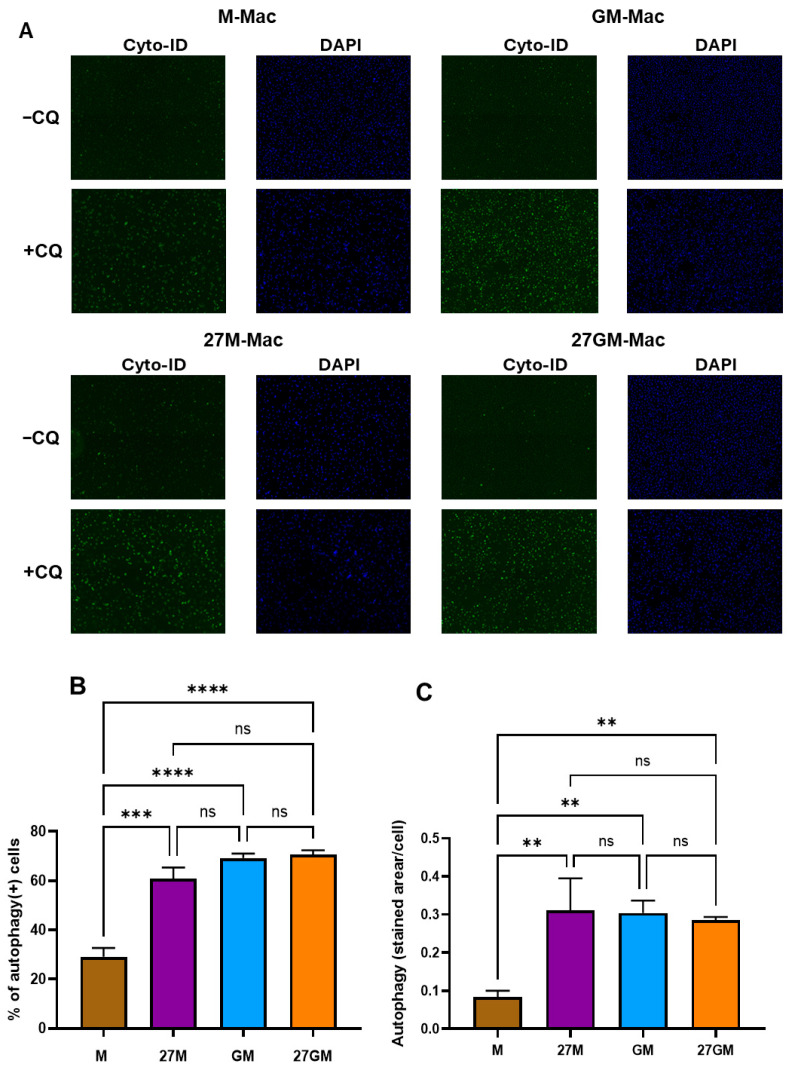
Comparison of autophagy activity. (**A**,**B**) M-Mac, 27M-Mac, GM-Mac and 27GM-Mac were induced in 96-well plates and then cultured overnight in D10 medium with or without 10 μM chloroquine (CQ). Autophagosome staining with CYTO-ID and nuclear stain with Hoechst 33342 were performed using the Cyto-ID autophagy detection kit as described in Section 2. (**A**) Stained cells without or with CQ were imaged with a Zeiss Axio Observer A1 motorized microscope at a 10× magnification. (**B**,**C**) Image analysis of stained cells with CQ was performed using the Fiji ImageJ software. For each channel, a background threshold was set up to create 8-bit masks. For the green channel, the total stained area was retrieved as a measure of autophagosome staining, while particle counts following watershed processing of the blue channel for Hoechst 33342 gave the corresponding cell number. (**B**) The percentage of cells indicating green color and (**C**) autophagy activity, calculated by the average stained area per cell, for each experimental condition of randomly selected 1000 cells from three independent donors. Data are presented as mean ± SE (*n* = 3). Statistical analysis was conducted using One-way ANOVA. **: *p* < 0.01, ***: *p* < 0.001, ****: *p* < 0.0001, ns: not significant.

IL-27 promoted autophagy in human AB serum-differentiated macrophages [28], but the effects of IL-27 on autophagy promotion in M-Mac and GM-Mac have not been investigated. Therefore, we compared autophagic activity among the four subsets (M-Mac, 27M-Mac, GM-Mac and 27GM-Mac). Cells were stained with Cyto-ID in the presence of chloroquine (CQ), and autophagy-promoting activity was assessed by fluorescence microscopy. This assay measures accumulated autophagosome/autolysosome and does not directly assess autophagic flux. To quantify the proportion of cells exhibiting autophagy, 1000 cells were counted per condition, and CQ-dependent autophagy activity was quantified as the stained area per cell, as described previously [28]. GM-Mac exhibited 2.4 ± 0.1-fold higher basal autophagy activity than M-Mac (*n* = 3, *p* < 0.001). An effect of IL-27 on autophagy was observed only in 27M-Mac (Figure 13A). Specifically, IL-27 treatment of M-Mac, but not GM-Mac, increased the percentage of cells exhibiting autophagy by 2.1 ± 0.15-fold (*n* = 3, *p* < 0.001) compared with M-Mac and increased autophagy activity by 3.7 ± 0.5-fold (*n* = 3, *p* < 0.01) in 27M-Mac relative to M-Mac (Figure 13B,C). Thus, in terms of autophagy-promoting activity, 27M-Mac possessed autophagy activity comparable to that observed in GM-Mac and 27GM-Mac. And IL-27 treatment only enhanced autophagy in M-Mac, not GM-Mac.

#### 3.6.2. Potential Mechanism Underlying Autophagy Regulation

**Figure 14:**
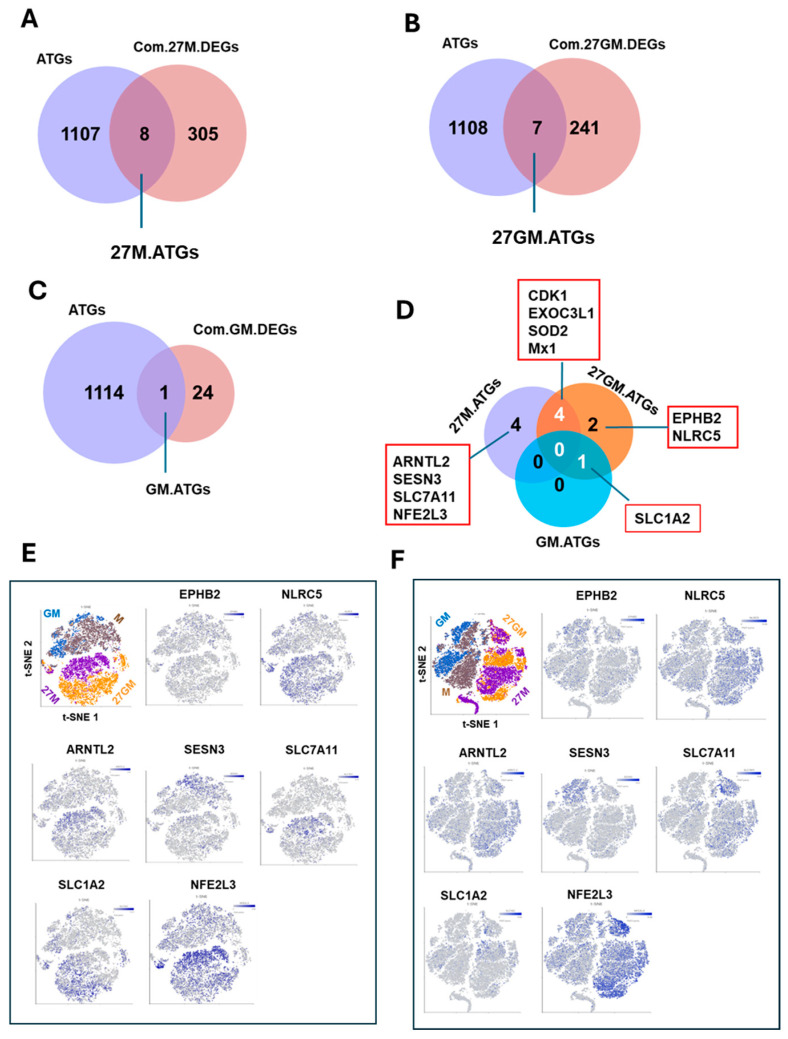
Identification of autophagy-associated genes (ATGs) in each DEG. (**A**–**C**) ATGs in Com.27M.DEGs: 27M.ATGs(**A**), ATGs in Com.27GM.DEGs: 27GM.ATGs (**B**), and ATGs in Com.GM.DEGs: GM.ATGs (**C**) were identified by Venn diagram analyses. (**D**) To identify common ATGs among 27M.ATGs, 27GM.ATG and GM.ATG, a Venn diagram analysis among 27M.DEGs, 27GM.DAGs and GM.DAGs were performed. The names of overlapped or non-overlapped genes are shown in red boxes. (**E**,**F**) The t-SNE plots indicate each gene distribution in Donor (**E**) and Donor B (**F**). Blue dots indicate cells expressing the indicated gens, while gray dots represent cells lacking the expression of the gene. The color intensity indicates relative gene expression levels, with gray indicating low or absent expression and darker blue indicating higher expression as shown in color bars.

To elucidate mechanisms underlying enhanced autophagy in 27M-Mac and to identify genes potentially associated with autophagy, Venn diagram analysis was carried out using the 313 common 27M.DEGs (Figure 3A) with 1115 human autophagy-related genes (ATGs) curated in the Autophagy Database (Supplementary Table S4; https://autophagy.info/download/download.html, accessed on 21 March 2025). This analysis identified eight ATGs within the 27M.DEG set (Figure 14A; Supplementary Table S9). Similarly, we intersected the 248 common 27GM.DEGs (Figure 3B) and the 25 common GM.DEGs (Figure 3C) with ATGs, identifying seven and one overlapping genes, respectively (Figure 14B,C; Supplementary Table S9). To determine whether any ATGs were shared among 27M-Mac, 27GM-Mac, and GM-Mac, we performed a Venn diagram analysis using the 27M.ATG, 27GM.ATG, and GM.ATG gene sets. No DEGs were common to all three subtypes (Figure 14D), suggesting that the mechanisms regulating autophagy differ across these cell types. Four genes (*CDK1*, *EXOC3L1*, *SOD2*, and *MX1*) overlapped between 27M-Mac and 27GM-Mac, whereas *SLC1A2* was shared between GM-Mac and 27GM-Mac. The *SLC1A2* gene product may contribute to basal autophagy in these cells. As IL-27 enhanced autophagy in 27M-Mac, four genes in the 27M.ATG (*ARNTL2*, *SESN3*, *SLC7A11*, and *NFE2L3*) were considered candidates mediating this induction. In scRNA-Seq data, three of these genes (all except *SESN3*) were induced ≥3-fold in both Donor A and Donor B (Supplementary Table S13). Autophagy was detected in >60% of 27M-Mac. To assess cellular distribution, expression of each candidate gene was examined by t-SNE analysis for Donor A (Figure 14E) and Donor B (Figure 14F) (Supplementary Table S14). Notably, *NFE2L3* (nuclear factor erythroid 2-related factor 3) was expressed in 50% to 70% of 27M-Mac. Since *NFE2L3* encodes a transcription factor, it is unlikely to directly regulate autophagy; however, it may promote autophagy by inducing downstream ATG expression in 27M-Mac.

## 4. Discussion

In the present study, we compared the effects of IL-27-mediated polarization of M-Mac and GM-Mac by assessing anti-HIV activity, ROS induction, phagocytosis, autophagy, cell surface marker expression, and gene expression profiles by scRNA-Seq. As shown in Table 1 and summarized in Table 2, 27M-Mac and 27GM-Mac exhibited transcriptional and phenotypic similarity, despite modest differences across select functional assays (blue-highlighted cells in Table 2).

**Figure 15:**
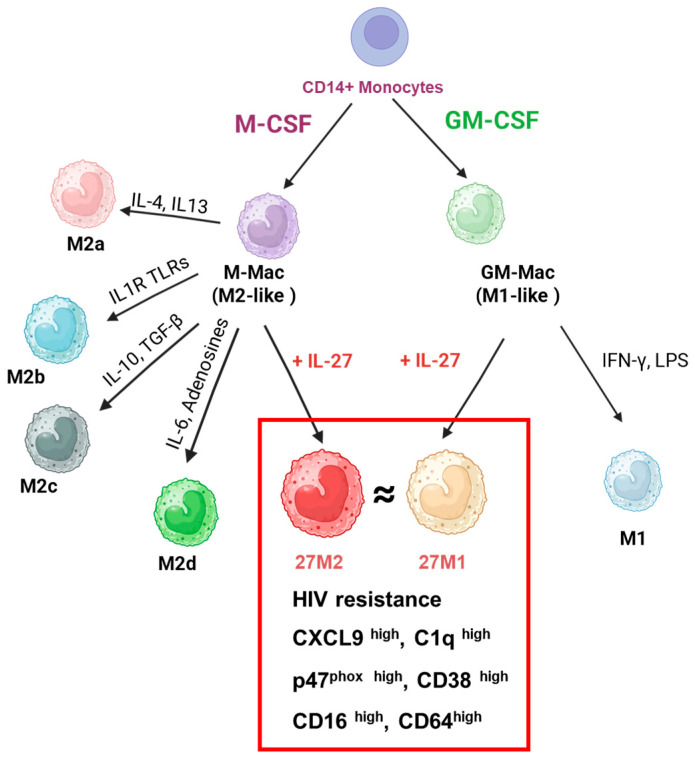
Diagram for 27M-Mac and 27GM-Mac. Diagram of canonical polarization and IL-27-mediated polarization of M-Mac (M2-like macrophages) and GM-Mac (M1-like macrophages). M1-like cells are polarized to M1 by IFN-γ, IFN-γ + LPS [57,58,59,60,61] or by Th1 cytokine stimulation [57,62]. M2-like cells are known to be polarized to M2a by IL-4 or IL13 [53,56,64,65,66], M2b by IL1R or TLRs, M2c by IL-10 or TGF-β, and to M2d by IL-6 or adenosines [41,67,68,69]. Following polarization by IL-27, both M-Mac and GM-Mac converge toward a subset characterized by a high level of CD38, CD16 and CD68 (CD38^hi^/CD16^hi^/CD64^hi^), and CXCL9 secretion with HIV resistance. Common biological functions assessed in this study were listed in the red box. This figure was created with BioRender, https://bioRender.com (accessed on 23 February 2025).

Although M-Mac and GM-Mac exhibit distinct baseline phenotypes, IL-27 stimulation for 3 days activates overlapping STAT1- and STAT3-dependent signaling pathways, followed by the induction of partially overlapping DEGs. At the phenotype level, the expression levels of CD16, CD38, and CD64 become more similar, and cellular function, including the anti-HIV activity, phagocytosis, autophagy, and ROS induction, is comparable between the two polarized cell types. Therefore, these transcriptional and phenotypic findings support the conclusion that IL-27 promotes convergence of M-Mac and GM-Mac toward a shared macrophage subtype (Figure 15).

Our analysis revealed some donor-specific variability in DEG profiles, with certain DEGs unique to individual donors and differences in expression magnitude even among shared DEGs. These differences can influence the magnitude of biological functions associated with the single-cell level. Nevertheless, the overall trends in the analyzed averaged function were consistent across donors, suggesting that the observed convergence reflects generalizable biological patterns rather than donor-specific artifacts. These findings highlight both the robustness of the functional trends and the importance of considering donor variability in transcriptomic studies.

M-CSF-differentiated MDMs (M2-like) can be polarized toward M2a, M2b, M2c, or M2d with appropriate stimulants, whereas GM-CSF-differentiated MDMs (M1-like) can be polarized toward classically activated M1 by a high concentration of IFN-γ or IFN-γ plus LPS stimulation [57,58,59,60,61]. Although IL-6 induces M2d [41,67,68,69] and IL-27 shares gp130 with IL-6, IL-27-polarized M-Mac was distinct from M2d [27]. It was considered the possibility that 27M-Mac may resemble M1. However, the current study shows that 27M-Mac is most similar to 27GM-Mac. In preliminary experiments, 100 ng/mL of IFN-γ or a combination of 20 ng/mL IFN-γ plus 20 ng/mL of LPS-induced M1 macrophages were functionally distinct from 27M-Mac and 27GM-Mac. Specifically, the IFN-γ–induced M1 macrophages showed approximately 5-fold higher PMA-stimulated ROS induction, nearly 3-fold higher CD38 (an M1 marker) expression and approximately 5-fold higher C1q (an M3 marker) production compared with 27M-Mac and 27GM-Mac. Comparing the results of the current study with the observations from the preliminary studies suggests that 27M-Mac and 27GM-Mac represent either partially polarized M1 and M3 macrophages or a distinct macrophage subtype. Further characterization is needed to define these states and their relationship to canonical polarized cells. The effects of IL-27 on other macrophage subsets (for example, M2b, M2c, or M3) also remain to be determined and may clarify whether IL-27 facilitates macrophage plasticity in vitro. In vivo mouse studies indicate that IL-27 augments M1 induction and alters the M1/M2 ratio [82,83]. Our findings may explain a mechanism in the skewed ratio, since IL-27 can directly shift M2-like macrophages toward an M1-biased or M1-associated profile.

HIV host factors, including dependency and restriction factors [84], have been extensively defined in HIV-susceptible cell types using siRNA, shRNA, and CRISPR screening approaches. In the present study, scRNA-Seq was performed using cells from two donors, which limits the generalizability of individual gene expression patterns and precludes definitive mechanistic conclusions. Nevertheless, t-SNE visualization (Figure 4E,F) indicated that individual cells displayed heterogeneous expression of host factor genes following IL-27 polarization; thus, a gold standard mechanism for anti-HIV activity may not exist in primary macrophages. These results suggest that antiviral mechanisms differ across individual macrophage subsets. Because 27M-Mac significantly suppressed HIV replication, with residual activity comparable to GM-Mac, we hypothesized that endogenous genes in GM-Mac contribute to HIV suppression and may also be induced in 27M-Mac. Venn diagram analysis identified MT1G and MT1H as genes meeting these criteria (Figure 4D). MT1G and MT1H are members of the metallothionein family [85], and contribute to metal ion (zinc and copper) homeostasis and protection against oxidative stress by scavenging ROS. Because oxidative stress has been reported to suppress HIV-1 replication [86], induction of MT1G and MT1H in cells may counteract the anti-HIV effect by buffering ROS. Therefore, despite their upregulation in 27M-Mac, MT1G and MT1H are unlikely to be the primary mediators of the anti-HIV activity. A total of 21 anti-HIV/host factor genes were identified as differentially expressed genes in 27M-Mac. The t-SNE plots demonstrated that *APOBEC3A* was uniquely and strongly induced in IL-27-treated cells compared to other cell types, as reported by others [17] and APOBEC3A functions as an anti-HIV in macrophages [87]. As demonstrated in Supplementary Table S8, 21 to 38% of 27M-Mac expressed APOBEC3A, yet suppressed HIV by over 90%, thereby indicating a potential collaborative effect between APOBEC3A and other factors in the suppression of HIV in 27M-Mac. The heterogeneity in the anti-viral gene expression suggests that mechanisms of antiviral effect may vary across individual macrophages, and antiviral activity in IL-27-polarized macrophages likely reflects cooperative or redundant effects among multiple host factors rather than reliance on a single gene product. Consequently, the efficacy of viral suppression in 27M-Mac (and 27GM-Mac) may extend beyond HIV to other viruses, including RNA viruses (e.g., influenza, SARS-CoV-2, and hepatitis C virus) and DNA viruses (e.g., hepatitis B virus, herpes simplex virus, and cytomegalovirus). However, the expression of multiple genes encoding antiviral and host-restriction factors in the cells represents correlative transcriptional associations, not mechanistic causality, at the level of individual genes. Further study is necessary for validation and to elucidate the underlying mechanisms.

27M-Mac and 27GM-Mac produced high amounts of ROS upon PMA stimulation. Because ROS was detected only following PMA stimulation and was accompanied by increased expression of p47^Phox^, these findings suggest that IL-27 primes macrophages for enhanced inducible NADPH oxidase–associated ROS production. The ROS-inducing activity was measured by hydrogen peroxide accumulation in culture supernatants using the Amplex Red assay. Therefore, the precise cellular source of ROS was not directly dissected in this study. Further studies will be required to directly distinguish the ROS origin.

Although FcγR expression was increased in 27M-Mac and 27GM-Mac, phagocytic activity in 27M-Mac and 27GM-Mac was reduced to 35% (*p* < 0.01) and 23% (*p* < 0.001) of that in M-Mac, respectively (Figure 10A,B). FcγR–mediated phagocytosis requires downstream signaling through Immunoreceptor Tyrosine-based Activation Motifs (ITAM)—Spleen tyrosine kinase (Syk)—Phosphoinositide 3-kinase (PI3K) pathways Crowley [88,89] and actin cytoskeletal remodeling [88,90]. Our findings suggest that IL-27-polarization affects post-receptor events rather than Fcγ receptor availability. IL-27 constitutively retained STAT1 and STAT3 activation (Supplementary Figure S1C), which may induce inhibitory regulators that attenuate Syk/PI3K signaling and limit actin-dependent phagocytic cup formation. Among DEGs associated with phagocytosis, *DYSF* (a negative regulator of phagocytosis) and *RAB7B* (a positive regulator of phagocytosis) were shared between 27M-Mac and 27GM-Mac. Population composition analysis of the scRNA-Seq data demonstrated that 21–26% of 27M-Mac and 19–33% of 27GM-Mac expressed *DYSF* (Supplementary Table S7). In contrast, *RAB7B* is expressed in approximately 20% of 27M-Mac and 27GM-Mac. Notably, scRNA-Seq indicated that nearly 60% of 27M-Mac and 27GM-Mac lacked detectable expression of both genes (Supplementary Table S7), whereas >90% of 27M-Mac and >80% of 27GM-Mac displayed phagocytic activity. These findings suggest that DYE and RAB7B may not be directly involved in the decreased phagocytosis. GM-Mac also exhibited lower phagocytic activity than M-Mac. *SPON2* was the only phagocytosis-associated gene identified in GM-Mac DEGs, and it encodes an extracellular matrix protein that regulates Fc-independent phagocytosis [89,91]. Additional factors may likely contribute to the reduced phagocytic activity of GM-Mac. Since CD14/TLR4-mediated phagocytosis in 27M-Mac and 27GM-Mac was also suppressed, these findings suggest that post-receptor events involved in the phagocytic process are impaired in the cells. Whether these suppressive effects reflect a shared downstream mechanism—such as altered cytoskeletal dynamics or signaling uncoupling—or distinct impacts on individual uptake pathways remains to be determined. Future studies will be required to define the contribution of alternative phagocytic routes, including scavenger receptor– and C-type lectin–mediated phagocytosis [92,93], in IL-27–polarized macrophages, to elucidate the molecular mechanism of the observed paradox between increased receptor expression and decreased phagocytosis. In the current study, we focused on comparing phagocytic activity, defined as bacterial internalization, rather than bactericidal activity. Evaluation of bactericidal function in IL-27–polarized cells may require further investigation.

Autophagy assays showed that endogenous autophagy in GM-Mac was 3.7 ± 0.23-fold higher than in M-Mac. Thus, although M-Mac and GM-Mac are often described as naïve or resting macrophages, their baseline functional states differ with respect to HIV replication, phagocytosis, and autophagy. These differences may be governed by GM-associated transcriptional profiles, including the 27GM.DEG set. In our previous study, IL-27 treatment enhanced autophagy in human AB serum-induced macrophages (AB-Mac) [28]. The present study extends these observations by showing that IL-27 differentially modulates autophagy in M-Mac and GM-Mac. Venn diagram analysis of autophagy-related gene sets (27M.ATGs, 27GM.ATGs, and GM.ATGs) revealed no genes shared among all three cell types, suggesting that autophagy promotion in 27M-Mac is regulated differently from the elevated basal autophagy observed in GM-Mac and 27GM-Mac. Consistent with this interpretation, four genes uniquely induced in 27M-Mac (*ARNTL2*, *SLC7A11*, *SESN3*, and *NFE2L3*) may contribute to autophagy promotion. IL-27-enhanced autophagy in AB-Mac is induced through an LC3- and mTOR-independent mechanism [28]. Therefore, the mechanism of driving enhanced autophagy in 27M-Mac may be similar.

In summary, this study shows that IL-27 polarizes M-Mac and GM-Mac toward a similar macrophage subset. These IL-27-polarized macrophages secrete CXCL9 and C1q and exhibit high expression of CD38, CD16, and CD64. In the present study, scRNA-Seq was performed using cells from two donors, which limits the generalizability of the inferred gene expression profiles. In addition, macrophage phenotypes were analyzed after three days of polarization. Although anti-HIV activity persisted for up to 14 days under the experimental conditions, the stability of the polarization state following cytokine withdrawal was not systematically evaluated across all functions and transcriptional levels. Therefore, the long-term stability of the IL-27–induced macrophage state remains to be defined. Further characterization of these IL-27–induced polarization states will advance understanding of macrophage plasticity and clarify the role of IL-27 in macrophage biology.

## 5. Conclusions

The current study demonstrates that IL-27 induces “convergent polarization” in M-CSF- and GM-CSF-induced primary MDMs toward a transcriptionally and phenotypically similar subset with shared antiviral and immunoregulatory features. IL-27–polarized macrophages suppress HIV replication, and exhibit increased expression of CD14, CD16, CD38, and CD64, secretion of CXCL9 and C1q, and coordinated modulation of ROS production, phagocytosis, and autophagy promotion. Single-cell RNA-seq analysis revealed transcriptional heterogeneity, suggesting that IL-27–mediated antiviral activity reflects collaborative mechanisms across macrophage subsets. Despite limited donor numbers, these findings support a role for IL-27 in promoting macrophage plasticity beyond canonical polarization states. Further characterization of this IL-27–induced macrophage subsets may clarify its broader relevance in antiviral immunity and inflammatory regulation.

## Figures and Tables

**Table 1:** Comparison of DEGs numbers among four cell types.

		DEGs ^1^			
Donor	Cell Types	M	27M	GM	27GM
A	M	-	-	-	-
	27M	560	-	-	-
	GM	119	755	-	-
	27GM	398	219	264	-
B	M	-	-	-	-
	27M	406	-	-	-
	GM	52	454	-	-
	27GM	464	30	369	-

^1^: DEGs were selected with absolute fold changes equal to or greater than three and *p*-values < 0.05.

**Table 2:** Summary of the assessed activities among subtypes *.

Phenotypes	M-Mac	27M-Mac	GM-Mac	27GM-Mac
HIV replication	++++	+	+	+
ROS induction **	+	++	+	+++
Phagocytosis of				
opsonized *E. coli*	+++	+	+	+
un-opsonized *E. coli*	++	+	++	+
Autophagy	+	++	++	++
Expression of				
CD14	+	++	+	++
CD38	+	+++	+	++
CD80	+	+	+	+
CD86	+	+	+	+
CD163	+	+	+	+
CD206	+	+	+	+
CD209	+	++	++	++
CD16	+/−	+	+/−	++
CD32	+	++	+	++
CD64	+	++	+	++
Secretion of ^§^				
CXCL9	+/−	++	+	+
C1q	+/−	+	−	+

*: The activities in each phenotype in each cell type were subjected to a relative scoring analysis, with results compared to the corresponding activity in M-Mac Activity levels are defined as: ++++ = very high activity, +++ = high activity, ++ = moderate activity, + = low activity, +/− = marginal activity, − = no activity. **: PMA-stimulated ROS induction. ^§^: Macrophages were polarized in 6-well plates in the absence or presence of IL-27 for three days. Cell-free culture supernatants were collected, and then protein amounts were assessed using ELISA.

## Data Availability

The original contributions presented in this study are included in the article/Supplementary Materials. Further inquiries can be directed to the corresponding author. The sequencing data have been deposited in the SRA database at the National Center for Biotechnology Information. The SRA database accession numbers: SAMN52956008, SAMN52956009, SAMN52956010, SAMN52956011, SAMN52956012, SAMN52956013, SAMN52956014, SAMN52956015, SAMN52956016, SAMN52956017. SAMN52956018. SAMN52956019. SRA records will be accessible with the following link after 1 November 2026 at https://www.ncbi.nlm.nih.gov/sra/PRJNA1354435 (accessed on 30 October 2025).

## Supplementary Materials

The following supporting information can be downloaded at: https://www.mdpi.com/article/10.3390/cells15060528/s1.

## Abbreviations

The following abbreviations are used in this manuscript:

ATGs	autophagy-related genes
CQ	Chloroquine
DEGs	differentially expressed genes
DYSF	dysferlin
FBS	fetal bovine serum
FPR2	formyl peptide receptor 2
GM-Mac	GM-CSF-induced macrophages
GM-CSF	Granulocyte-macrophage colony-stimulating factor
GCH1	GTP cyclohydrolase 1
AB-Mac	human AB serum-induced macrophages
IFN-γ	IFN- gamma
IL-27R	IL-27 receptor
27GM-Mac	IL-27-polarized GM-Mac
27M-Mac	IL-27-polarized M-Mac
FcγRs	immunoglobulin G Fc receptors
IFN	interferon
ISGs	interferon-stimulated genes
IL-27	Interleukin 27
LRRK2	leucine-rich repeat kinase 2
LPS	lipopolysaccharide
M-CSF	Macrophage colony-stimulating factor
MMP8	matrix metalloproteinase 8
M-Mac	M-CSF-induced macrophages
MDMs	monocyte-derived macrophages
MOI	multiplicity of infection
NCF1	Neutrophil cytosolic factor 1
PBMCs	peripheral blood mononuclear cells
PMA	phorbol 12-myristate 13-acetate
PID1	phosphotyrosine interaction domain containing 1
QC	quality control
qRT-PCR	Quantitative RT-PCR
RIPA	Radioimmunoprecipitation assay
RAB7B	Ras-related protein B
ROS	reactive oxygen species
SELENOP	selenoprotein P
scRNA-Seq	single-cell RNA sequencing
SOD2	superoxide dismutase 2
SNCA	synuclein alpha
t-SNE	t-distributed stochastic neighbor embedding
TGF-β	transforming growth factor β
TLR4	Toll-like receptor 4
WB	Western blotting
