# Supplementary material for: Integrated Functional and scRNA-Seq Analyses Reveal Convergence of M-CSF– and GM-CSF–Derived Macrophages Following IL-27 Polarization
Source: Cells. 2026 Mar 16;15(6):528. doi: 10.3390/cells15060528 (PMC13025208; doi:10.3390/cells15060528)
Supplement: Supplementary file 1 [file cells-15-00528-s001.zip › Supplemental fig for WB.pptx]

## Slide 1
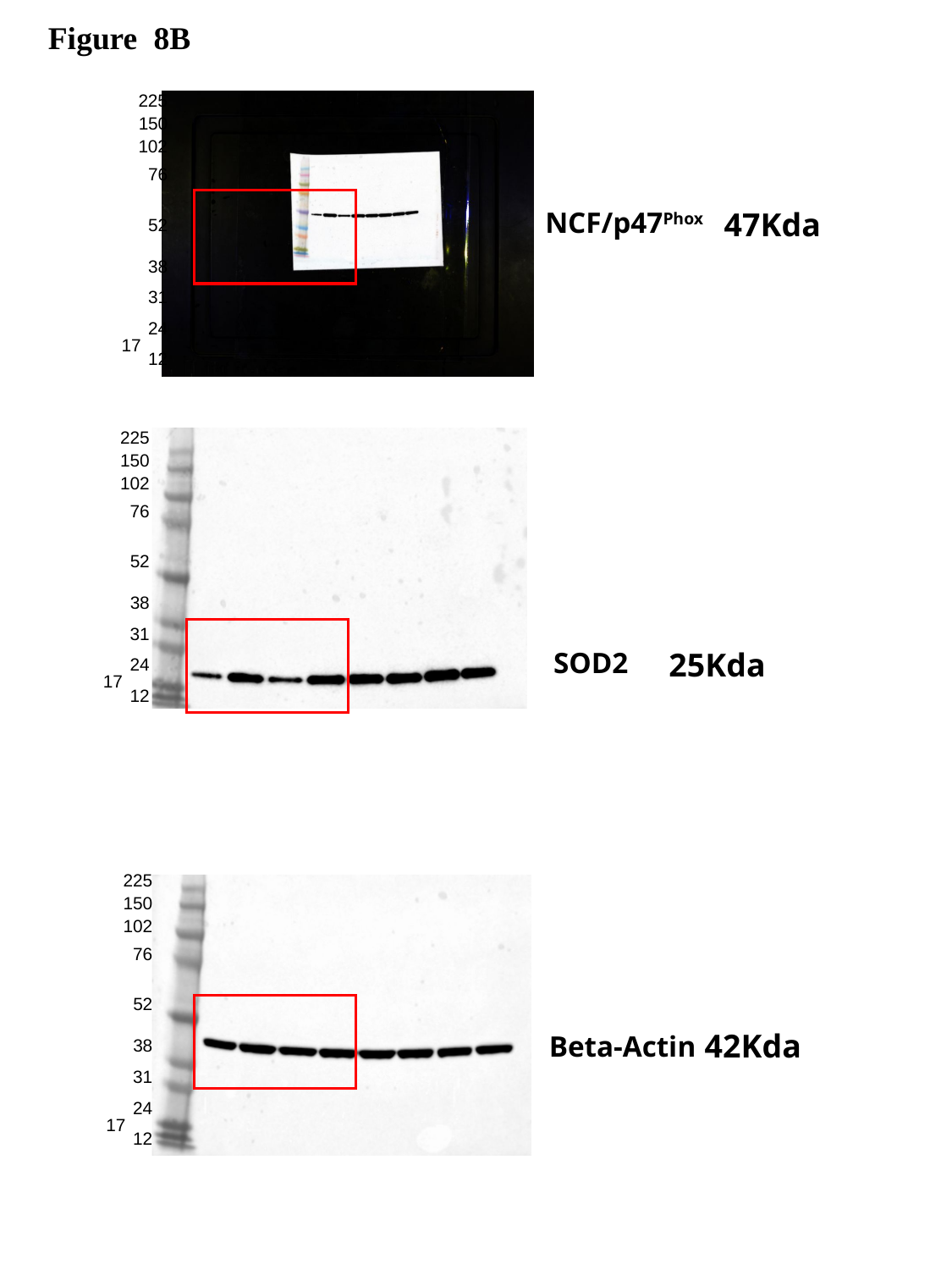

Figure 8B
225
150
102
76
52
38
31
24
17
12
NCF/p47Phox
47Kda
225
150
102
76
52
38
31
24
17
12
25Kda
SOD2
225
150
102
76
52
38
31
24
17
12
42Kda
Beta-Actin

## Slide 2
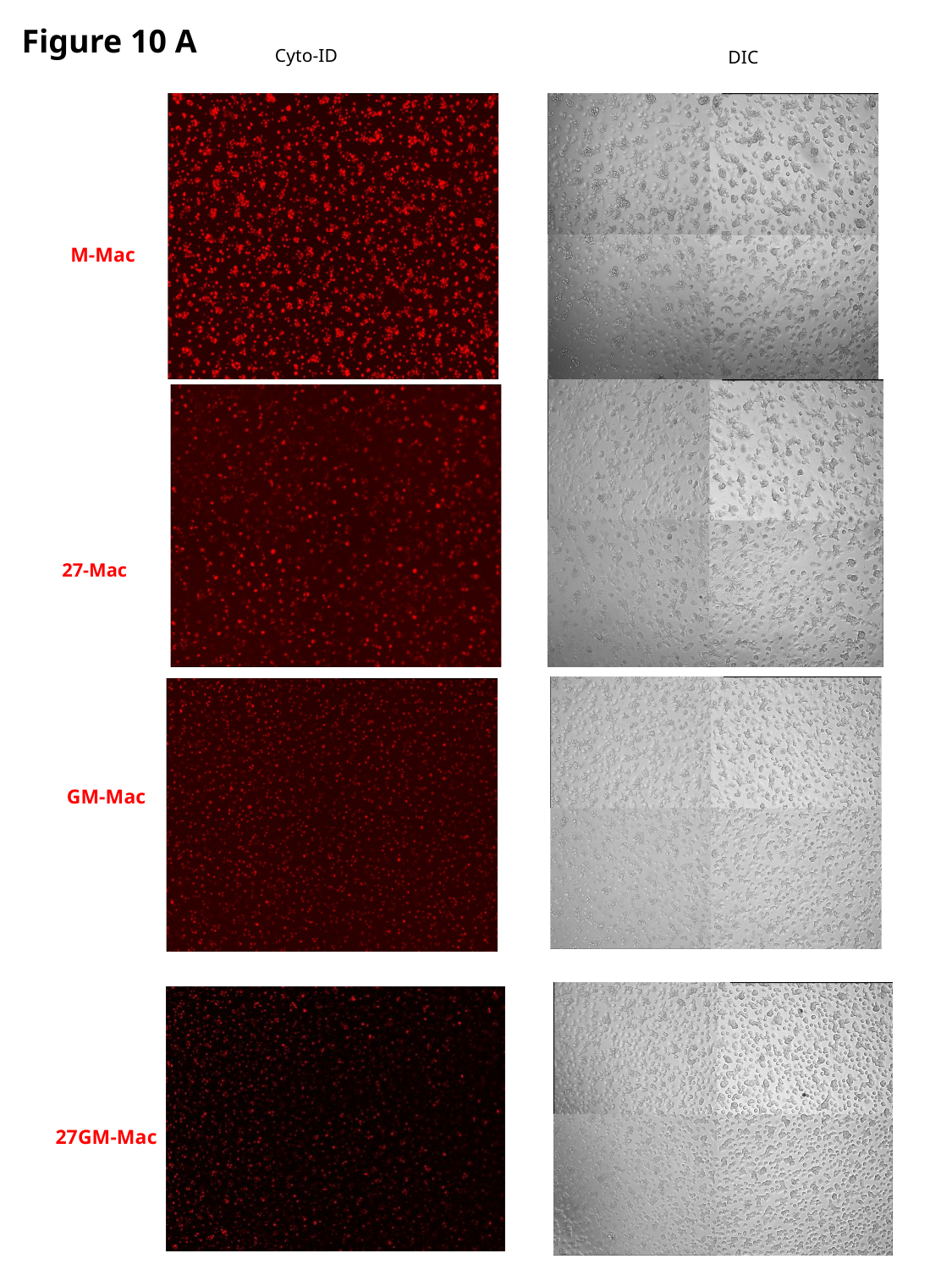

Figure 10 A
Cyto-ID
DIC
M-Mac
27-Mac
GM-Mac
27GM-Mac

## Slide 3
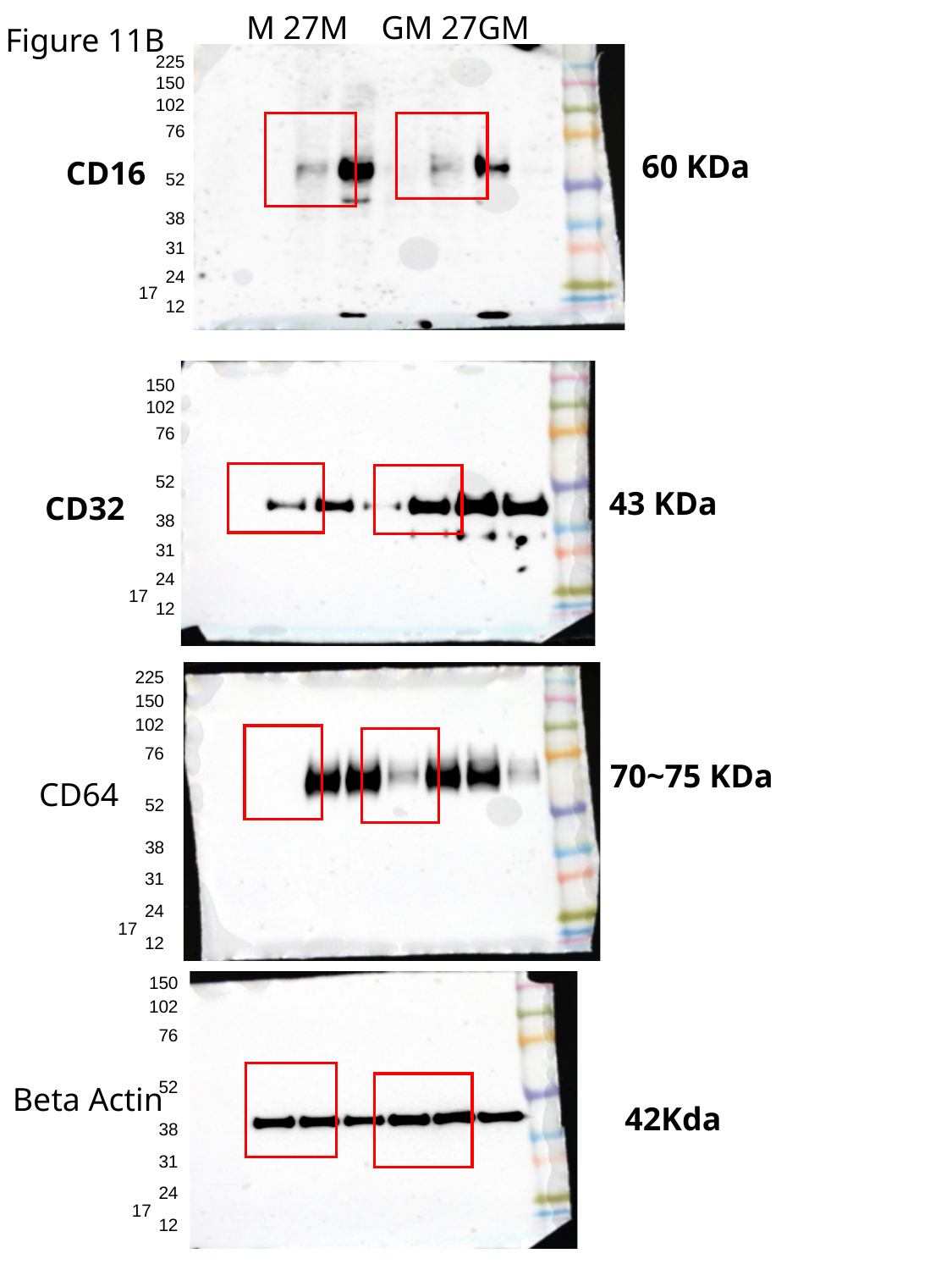

M 27M GM 27GM
Figure 11B
225
150
102
76
52
38
31
24
17
12
60 KDa
CD16
150
102
76
52
38
31
24
17
12
43 KDa
CD32
225
150
102
76
52
38
31
24
17
12
70~75 KDa
CD64
150
102
76
52
38
31
24
17
12
Beta Actin
42Kda

## Slide 4
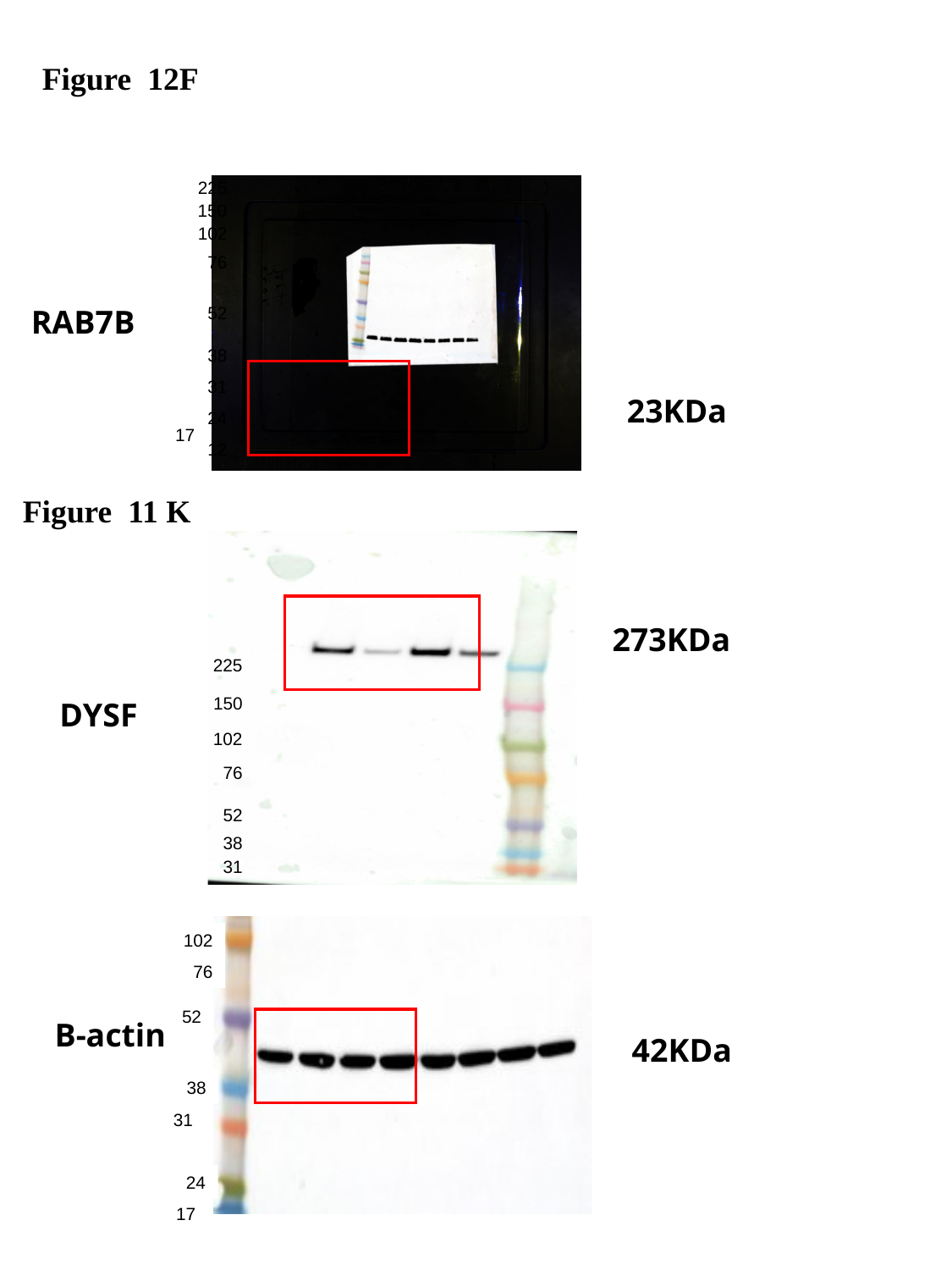

Figure 12F
225
150
102
76
52
38
31
24
17
12
RAB7B
23KDa
Figure 11 K
273KDa
225
150
DYSF
102
76
52
38
31
102
76
52
B-actin
42KDa
38
31
24
17

## Slide 5
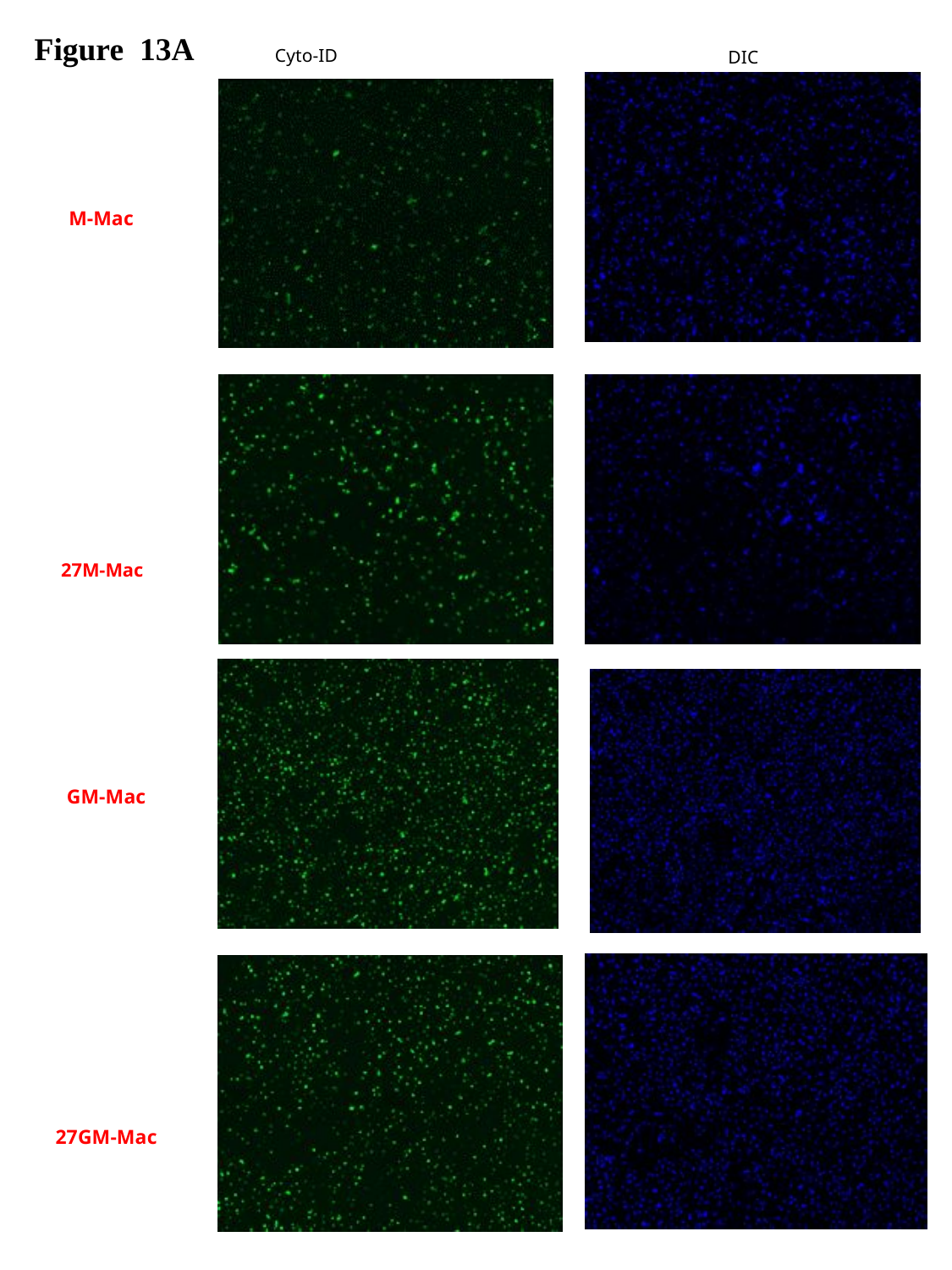

Figure 13A
Cyto-ID
DIC
M-Mac
27M-Mac
GM-Mac
27GM-Mac
