# Supplementary material for: Integrated Functional and scRNA-Seq Analyses Reveal Convergence of M-CSF– and GM-CSF–Derived Macrophages Following IL-27 Polarization
Source: Cells. 2026 Mar 16;15(6):528. doi: 10.3390/cells15060528 (PMC13025208; doi:10.3390/cells15060528)
Supplement: Supplementary file 1 [file cells-15-00528-s001.zip › Supplementary Figures S1-S5.pdf]

Supplementary Figures

T. Imamichi et al.

Supplementary Fig S1

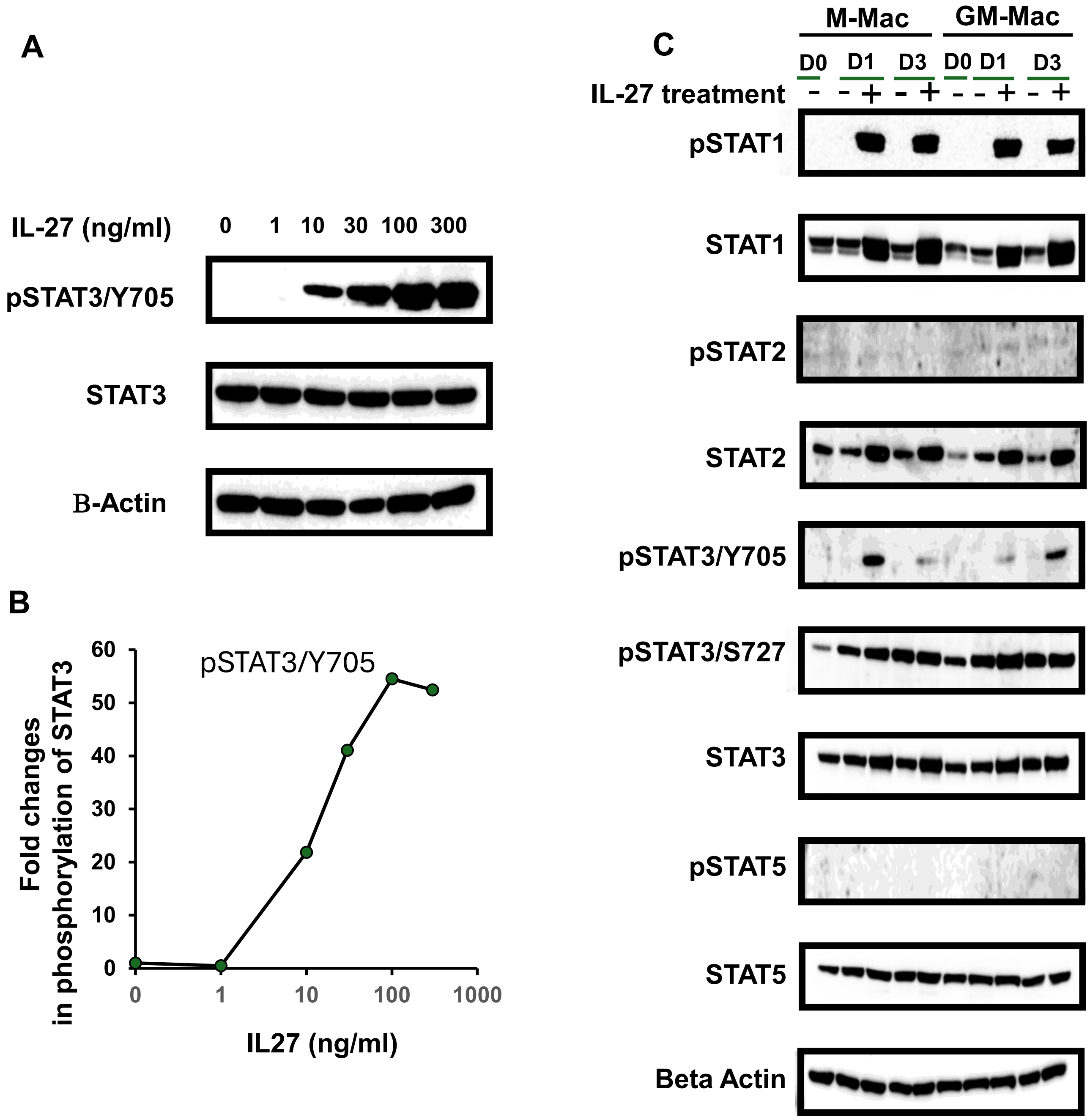

Supplementary Figure S1. STAT activation profile

(A) M-Mac were seeded at 1.5 x10^6 cells/ well in 6-well plates and then cultured with 0 to100 ng/mL of IL27 at 37°C for 15 min. The cell reaction was stopped by adding of cold PBS and then lysed with RIPA buffer in the presence of protease-phosphatase inhibitors. (B) The band intensity of each sample was analyzed using Fiji-ImageJ and then normalized by the band intensity of β-Actin and then the resulting values were plot as fold change compared to untreated cells. Results are representative of two independent experiments. (**C)** M-Mac and GM-Mac were seeded at 1.5 x10^6 cells/ well in 6-well plates and then cultured without or with 100 ng/mL of IL27 at 37°C for three days. Each time point at Day 0 (D0), Day 1 (D1), Day 3 (D3), cell reaction was stopped by adding of cold PBS and then lysed with RIPA buffer in the presence of protease-phosphatase inhibitors. WB was performed as described in the Materials and Methods.

S. Figure S2

A

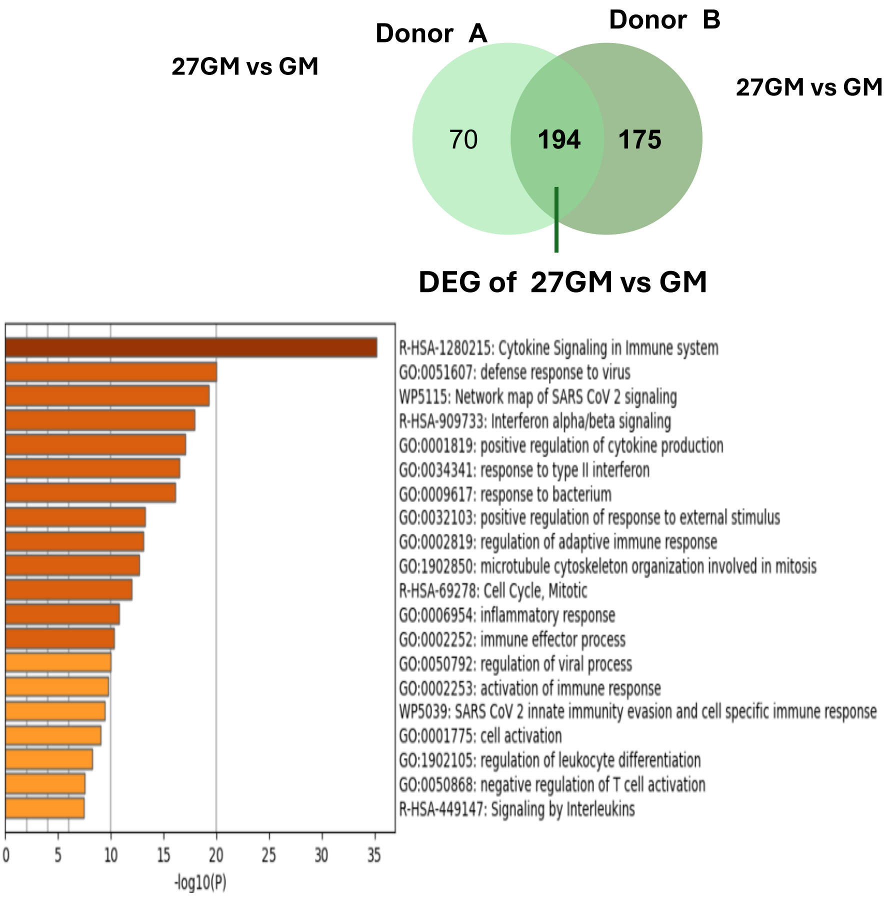

B

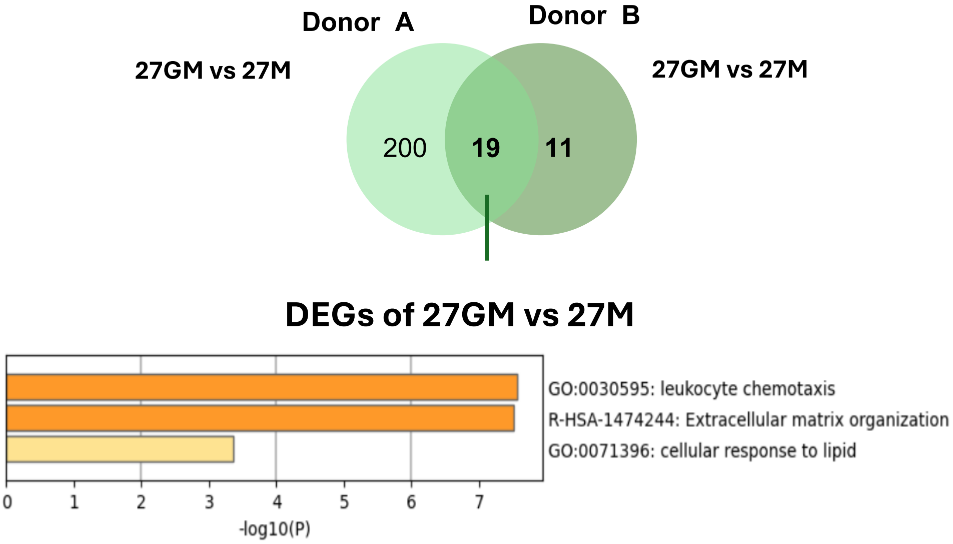

Supplementary Figure S2. common DEG and annotation analysis

To obtain common DEGs, each DEG from 27GM vs GM (A) and 27GM vs 27M (B) were subjected for Venn diagram analysis. Annotation analysis was conducted using gene list of 197 common 27GM vs. GM genes and 19 common 27GM vs. 27M genes.

S. Figure S3

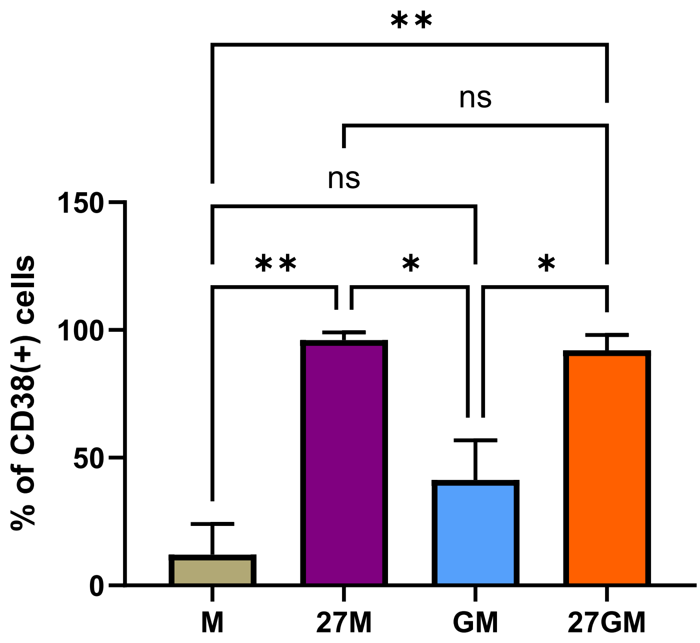

Supplementary Figure S3. The population of CD38-expressing (CD38+) cells

M- and GM-Mac were polarized in the absence or the presence of IL-27 for three days, and then CD38 expression was detected using FACS as described in Materials and Methods. Three independent donor cells were subjected, and the results of the CD38-positive cells were statistically analyzed. Data demonstrates means ± SE (n=3). *: $p<0.05$, **: $p<0.01$, ns: not significant.

S. Figure S4

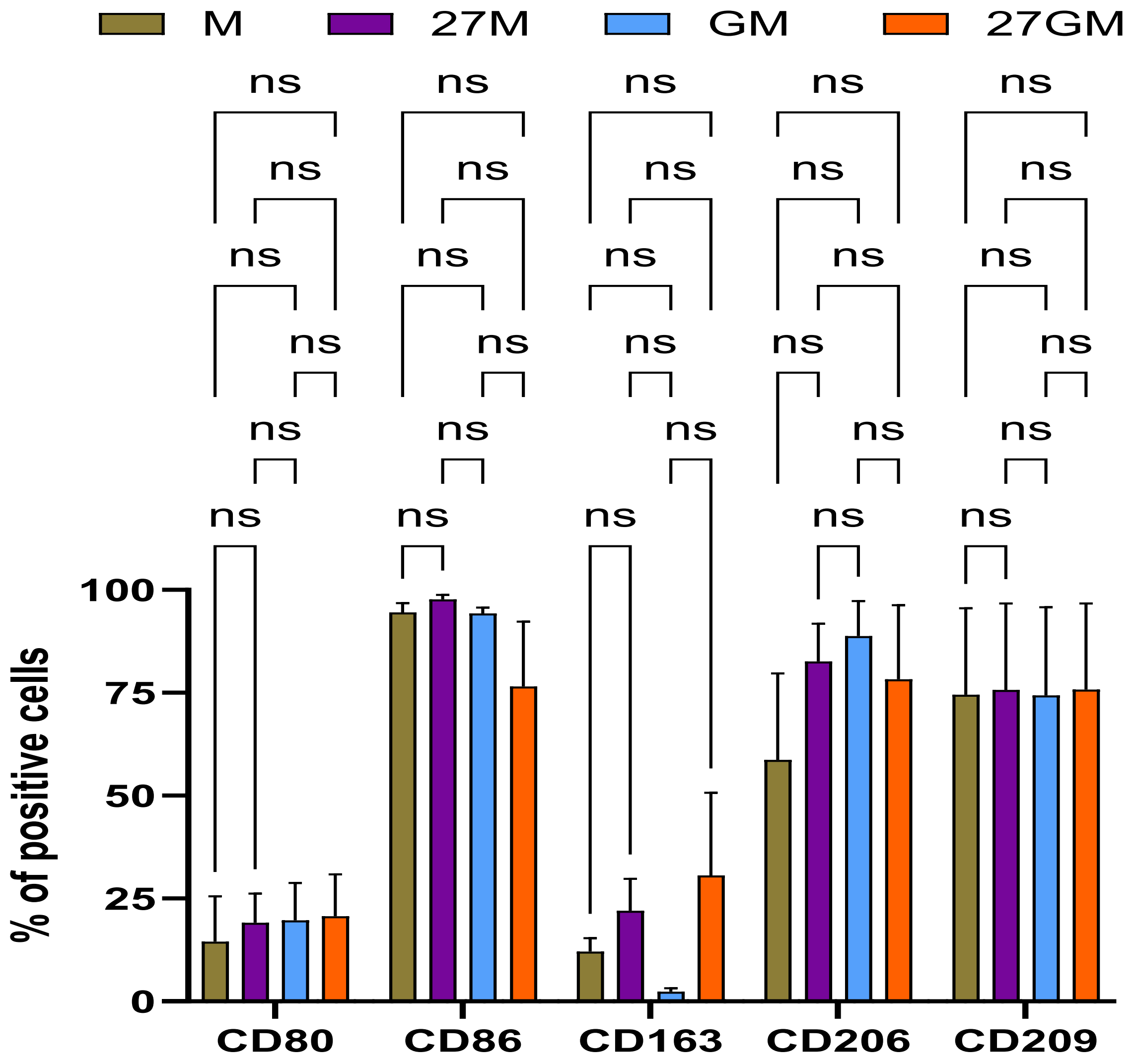

Supplementary Figure S4. The population of the cells expressing each macrophage markers.

M-Mac and GM-Mac were polarized with IL-27 for three days, and then CD80, CD86, CD163, CD206, and CD209 expression were detected using FACS as described in the Materials and Methods. Three independent donor cells were subjected, and the results of cells expressing each marker were statistically analyzed. Data demonstrates means ± SE (n=3). ns: not significant

S. Figure S5

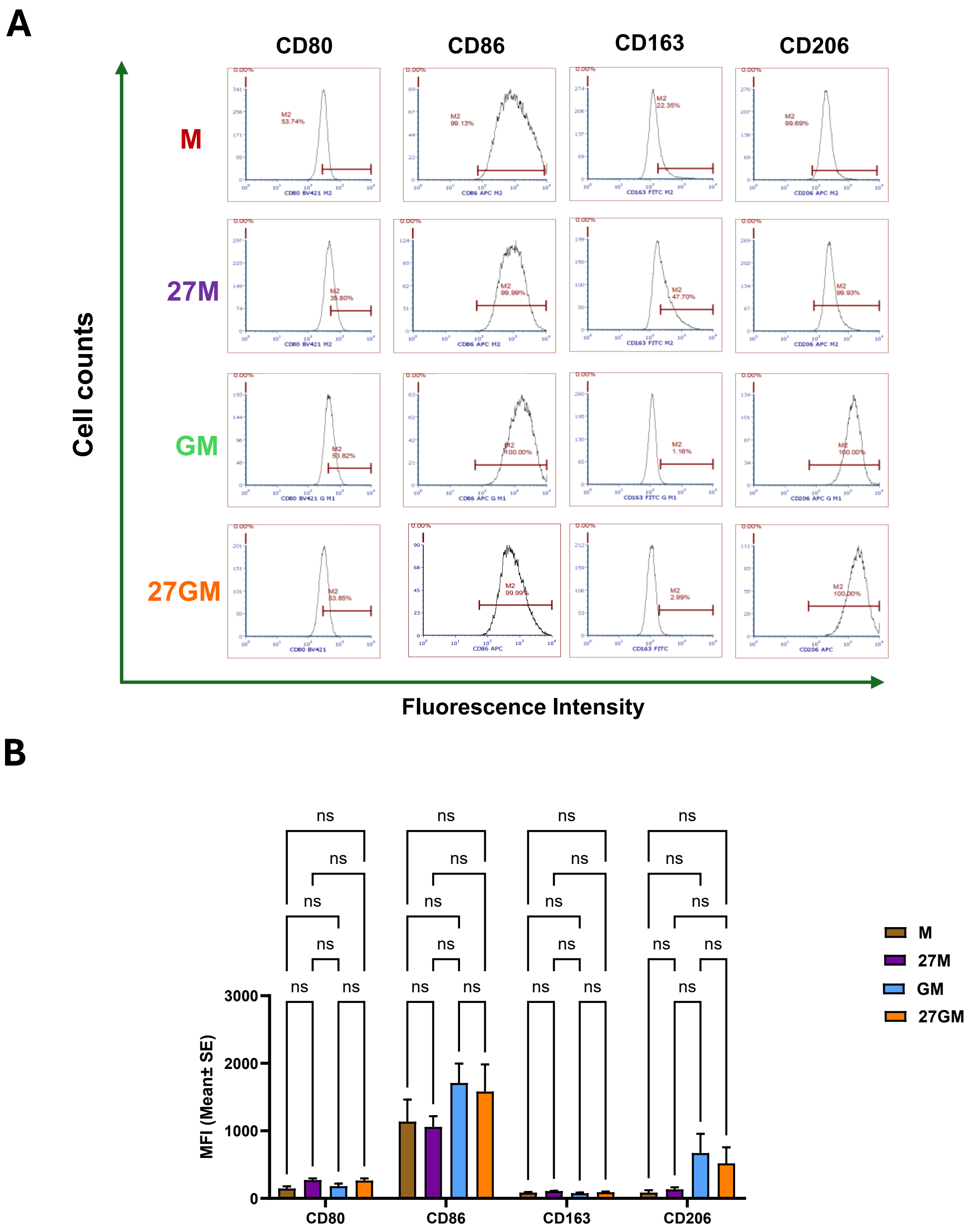

Supplementary Figure S5. Comparison of the expression of macrophage markers.

The expression of macrophages markers (CD80, CD86, CD163, and CD206) on M-Mac 27M-Mac, GM-Mac, and 27GM-Mac were compared using FACS as shown in Materials and Methods. Each isotype control was used to set positive gates shown using horizontal bars on histograms. (A) Results are representative of data from three independent experiments. (B) MFI calculated from three independent donors were calculated for each marker. Data are presented as mean ± SE Statistical analysis was conducted using Two-way ANOVA. ns: not significant.

S. Figure S6

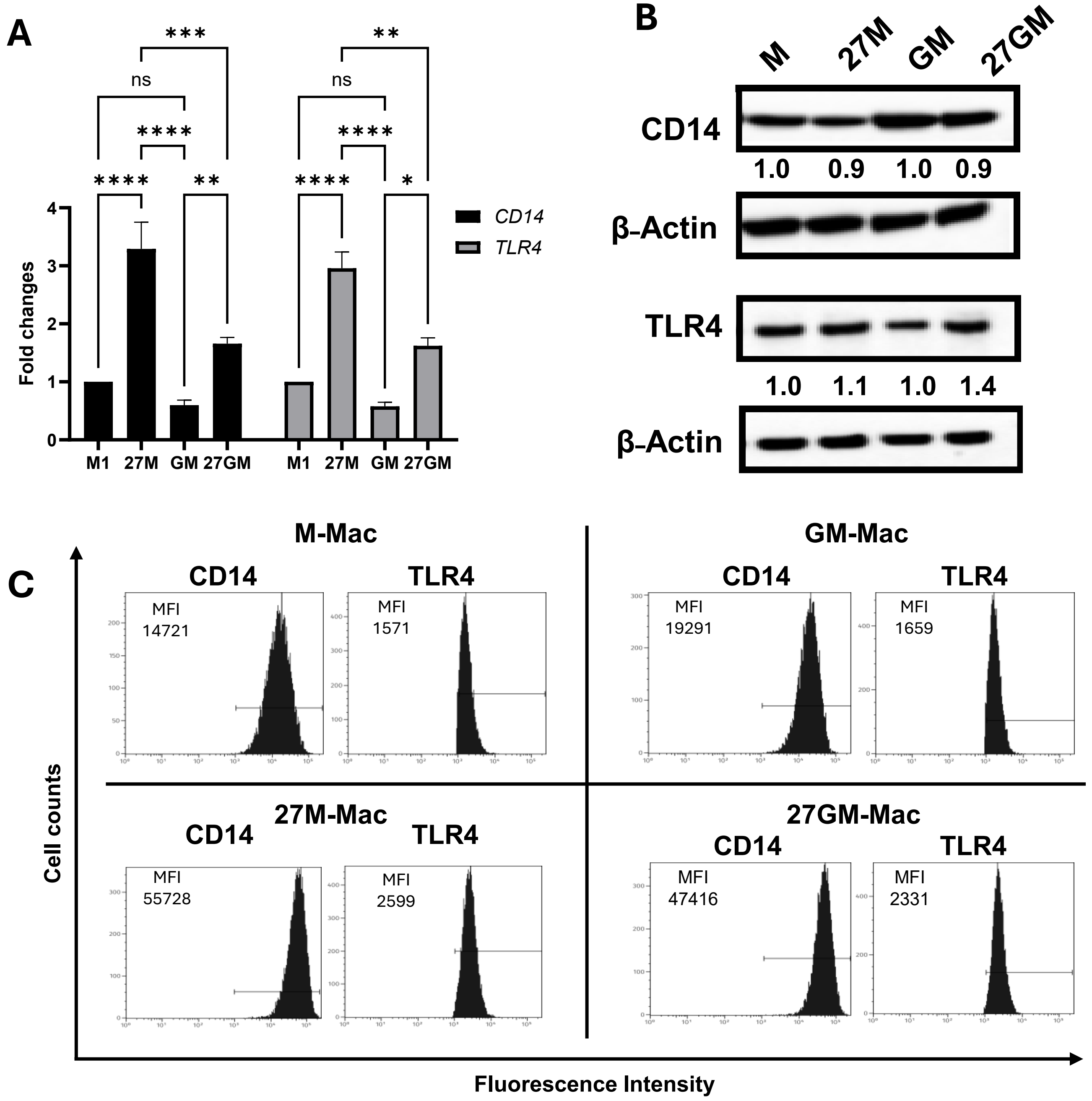

Supplementary Figure S6. Comparison of the expression of CD14 and TLR4.

(A) CD14 and TLR4 gene expression in M-Mac (M), 27M-Mac (27M), GM-Mac (GM) and 27GM-Mac (27GM) was assessed using qRT-PCR. Data are presented as mean ± SE from three independent donors. Statistical analysis was conducted using Two-way ANOVA.* : p< 0.05, **: p<0.01, *** : p<0.001, ****: p<0.0001, ns: not significant. (B) WB was performed using whole cell lysates from M, 27M, GM, and 27GM with anti-CD14 and anti-TLR4 antibodies. Data represents one of three independent donors. The band intensity of CD14 and TLR4 proteins was normalized by the band intensity of β-Actin (Image J), the values are indicated in the image.(C) The expression of CD14 and TLR4 on M-Mac, 27M-Mac, GM-Mac, and 27GM-Mac were compared using FACS as shown in Materials and Methods. Each isotype control was used to set positive gates. Results are representative of data from two independent donors.
